# Integrated Analysis of Serotonin-Related Biomarkers in Thoracic Aortic Aneurysm Based on Machine Learning and Single-Cell Sequencing Data

**DOI:** 10.3390/biomedicines14102218

**Published:** 2026-09-30

**Authors:** Shichao Zhu, Mieradilijiang Abudupataer, Hua Li, Zheng Zuo, Junyu Zhai, Tao Yan, Hongqiang Zhang, Hao Lai, Chunsheng Wang, Kai Zhu, Ben Huang, Nan Chen

**Affiliations:** Department of Cardiac Surgery and Shanghai Institute of Cardiovascular Diseases, Zhongshan Hospital, Fudan University, Shanghai 200032, China

**Keywords:** thoracic aortic aneurysm, serotonin, biomarker, machine learning, single-cell RNA sequencing

## Abstract

**Background:** Serotonin, as an important neurotransmitter and signaling molecule, can affect thoracic aortic aneurysm (TAA) by regulating vascular cell proliferation, angiogenesis, and immune status. This research aimed to conduct a comprehensive analysis to pinpoint serotonin-related biomarkers in TAA and to elucidate their potential molecular mechanisms. **Methods:** Biomarkers were selected and validated through differential expression analysis, weighted gene co-expression network analysis (WGCNA), machine learning algorithms, and expression verification. Moreover, the constructed nomogram, enrichment analysis, immune infiltration analysis, and compound prediction validated the regulatory roles of the biomarkers in TAA. The single-cell dataset was analyzed to determine key cells and perform pseudo-time analysis. Simulated knockout of biomarkers was performed to determine their underlying functions and related pathways. Finally, reverse transcription quantitative PCR (RT-qPCR) was employed to confirm the expression levels of the biomarkers. **Results:** Overall, two biomarkers (CD300A and CLU) were determined to be associated with serotonin in TAA. The nomogram showed good performance in predicting TAA risk. These biomarkers were significantly enriched in the hematopoietic cell lineage and natural killer (NK) cell mediated cytotoxicity. Biomarker expression was significantly correlated with the infiltration proportions of M2 macrophages and neutrophils. The biomarkers were stably bound to tetrachlorodibenzodioxin and bisphenol A. Moreover, pseudo-time analysis demonstrated a notable correlation between biomarker expression and the differentiation status of monocytes/macrophages/dendritic cells (MonoMac/DC). Genes perturbed by knockout were enriched in the pyrimidine metabolism and RIG-I-like receptor signaling pathway. Finally, RT-qPCR validated that CLU was downregulated and CD300A was upregulated in the TAA group. **Conclusions:** Biomarkers (CD300A and CLU) associated with serotonin in TAA were identified, with their expression trends validated by RT-qPCR. This work provides novel bioinformatic insights, and direct functional experiments are required to support future therapeutic development for TAA.

## 1. Introduction

Thoracic aortic aneurysm (TAA) is a life-threatening cardiovascular condition defined by permanent, localized dilation of the thoracic aorta, typically exceeding 50% of the normal vessel diameter, and is associated with a high risk of rupture or acute dissection if left untreated [1]. The disease affects approximately 6–10 per 100,000 individuals per year, predominantly older males, with an insidious clinical course that frequently escapes detection until catastrophic events occur [1]. The underlying pathophysiology involves a complex interplay of genetic susceptibility, hemodynamic stress, chronic inflammation, oxidative stress, and dysregulated extracellular matrix (ECM) remodeling [2]. Mutations in genes regulating the transforming growth factor-β (TGF-β) signaling cascade, such as *FBN1*, are particularly prevalent in hereditary syndromes including Marfan syndrome and Loeys–Dietz syndrome, which substantially elevate the risk of TAA onset [3]. At the cellular level, phenotypic switching of vascular smooth muscle cells (VSMCs) from a contractile to a synthetic state, coupled with macrophage-driven matrix metalloproteinase (MMP) secretion, contributes progressively to medial layer degeneration and aortic wall structural compromise [2]. Current clinical management relies on periodic imaging surveillance, blood pressure control, and elective surgical repair—either open surgery or thoracic endovascular aortic repair (TEVAR). However, both modalities carry significant limitations: TEVAR is associated with reintervention rates approaching 20–32% due to endoleaks and graft migration, while open repair entails elevated perioperative morbidity, particularly in patients with comorbidities [1]. Crucially, effective pharmacological agents to halt disease progression remain unavailable, rendering the identification of reliable biomarkers and druggable molecular targets a pressing clinical priority.

Serotonin (5-hydroxytryptamine, 5-HT) is a pleiotropic signaling molecule that exerts important regulatory effects on vascular biology beyond its well-established role as a central neurotransmitter. At the periphery, serotonin modulates vascular tone, platelet aggregation, and immune cell activation via a diverse repertoire of receptors—principally 5-HT2A, 5-HT2B, and 5-HT3—expressed on VSMCs, endothelial cells, and circulating immune cells [4]. Mounting evidence implicates the serotonergic system in aortic pathology: platelet-released serotonin promotes VSMC phenotypic switching, proliferation, migration, and pro-inflammatory cytokine (IL-6) secretion through MAPK and JAK2 signaling, thereby driving aortic wall remodeling [5]. Moreover, periaortic adipose tissue has been identified as a local serotonin reservoir that amplifies vascular inflammation through 5-HT3 receptor-mediated pathways, and pharmacological 5-HT3 antagonism markedly attenuates experimental aneurysm formation in murine models [6]. Serotonin signaling also intersects with immune regulation, influencing macrophage polarization and immune cell infiltration that collectively remodel the vascular microenvironment [4]. Additionally, dysregulated serotonergic signaling, including altered tryptophan hydroxylase 1 expression and facilitated serotonin release from periaortic fat, has been demonstrated to contribute to aortic aneurysm development in genetically susceptible mouse model [7]. Despite these mechanistic insights, which are largely from cellular and animal models, critical knowledge gaps remain for human TAA. First, human-aorta-derived serotonin-associated gene signatures and functionally validated biomarkers are lacking. Second, it remains unclear how serotonin-related molecules remodel the human aortic immune microenvironment and function during immune-cell differentiation at single-cell resolution. Most prior TAA transcriptomic biomarker work used unbiased genome-wide screening without focusing on pathogenic axes like serotonin signaling; few combined bulk and single-cell multi-omics, and only a small subset of signatures were validated in clinical specimens. To fill these gaps, we applied a serotonin-pathway-guided bioinformatic pipeline integrating WGCNA, multiple machine-learning algorithms, single-cell analysis, virtual gene-knockout simulation, molecular docking, and clinical RT-qPCR to identify and characterize serotonin-relevant human TAA biomarkers.

Single-cell RNA sequencing (scRNA-seq) has emerged as a transformative technology that enables unbiased transcriptomic profiling at single-cell resolution, resolving the cellular heterogeneity that bulk sequencing approaches inevitably obscure [8]. In the context of TAA, scRNA-seq has begun to redefine our understanding of the disease-associated cellular ecosystem, revealing diverse subpopulations of VSMCs, macrophages, monocytes, and dendritic cells that undergo distinct transcriptional reprogramming during aneurysmal progression [9]. In particular, proinflammatory macrophage and monocyte subsets have been shown to expand significantly within the TAA wall, engaging in profibrotic and cytotoxic intercellular communication that drives ECM degradation and VSMC apoptosis [9]. The integration of bulk transcriptomics with scRNA-seq data offers a multiscale perspective—identifying disease-relevant gene modules at the population level while pinpointing the precise cellular compartments mediating pathological effects—thereby substantially enhancing the translational value of bioinformatics discoveries [10]. In the present study, we performed a comprehensive bioinformatics analysis integrating bulk transcriptomic datasets with scRNA-seq data to identify and characterize serotonin-related biomarkers in TAA. Through a stepwise analytical pipeline combining differential expression analysis, weighted gene co-expression network analysis (WGCNA), five complementary machine learning algorithms, and extensive functional annotation, we identified two robust biomarkers—CD300A and CLU—whose expression was validated across independent cohorts and confirmed by RT-qPCR in clinical specimens. Downstream analyses including gene set enrichment analysis (GSEA), immune infiltration profiling, molecular docking, and single-cell-level virtual gene knockout experiments further illuminated the mechanistic contributions of these biomarkers to TAA pathogenesis. Together, our findings provide novel molecular insights into the serotonin–TAA axis and offer promising candidates for early diagnosis and targeted therapeutic intervention in TAA.

## 2. Materials and Methods

### 2.1. Preparation of Sequencing Data

Public transcriptome data from RNA sequencing (RNA-seq) datasets (GSE26155 and GSE202267) and the single-cell RNA sequencing (scRNA-seq) dataset GSE155468, all associated with thoracic aortic aneurysm (TAA), were retrieved from the Gene Expression Omnibus (GEO) database (http://www.ncbi.nlm.nih.gov/geo/, accessed on 24 April 2026). Among them, the training set GSE26155 (platform: GPL5175) contained data from 43 TAA and 43 normal aortic wall tissue samples. The validation set GSE202267 (platform: GPL24676) contained data from 4 TAA and 4 normal aortic wall tissue samples. The scRNA-seq dataset GSE155468 (platform: GPL24676) included 3 control groups and 8 TAA groups of human aortic tissue at the single-cell level. Moreover, serotonin-related genes (SRGs) (Table S1) were obtained from the Molecular Signatures Database (MSigDB) [11].

### 2.2. Differential Expression Analysis

Bulk-differentially expressed genes (DEGs) between TAA and control samples were screened using the limma package (v 3.56.2) [12] in GSE26155 (|log_2_ fold change (FC)| > 0.5 and adjusted *p* < 0.05, Benjamini-Hochberg). Afterwards, the ggplot2 package (v 3.5.2) and pheatmap package (v 1.0.12) were utilized to establish the volcano plot and heatmap, respectively, to display the markers and expression patterns of bulk-DEGs.

### 2.3. Weighted Gene Co-Expression Network Analysis (WGCNA)

In the training set GSE26155, the ssGSEA algorithm was performed using the GSVA package (v 1.53.28) [13] to demonstrate the differences in SRG scores between TAA and control samples (*p* < 0.05). The original curated serotonin-related gene set from MSigDB mainly contains genes directly participating in serotonin synthesis, transport and receptor function. Considering that serotonin signaling can trigger broad downstream transcriptional cascades in aortic tissue, we expanded the gene repertoire by incorporating co-expressed genes from modules highly correlated with ssGSEA-derived serotonin-pathway activity, which allows capturing downstream effector genes regulated by serotonin signaling, albeit some indirectly associated genes may be included. Subsequently, WGCNA was conducted on SRGs using the WGCNA package (v 1.73) [14]. First, sample clustering was performed using the hclust function, and the soft threshold was determined for the clustered samples using the pickSoftThreshold function to ensure that gene–gene interactions conformed to the scale-free network distribution to the greatest extent (R^2^ > 0.8). Based on the distance matrix calculated by the topological overlap matrix (TOM), hierarchical clustering of samples was performed using the hierarchicalCluster function to construct a hierarchical clustering dendrogram reflecting co-expression relationships. The key modules showing the most positive and most negative correlations with SRG scores were selected (|cor| > 0.3, *p* < 0.05), and the genes within these modules were defined as serotonin-related module genes.

### 2.4. Enrichment Analysis and Protein–Protein Interaction (PPI) Network

The ggvenn package [15] (v 0.1.10) (https://CRAN.R-project.org/package=ggvenn, accessed on 1 January 2024) was used to identify overlapping genes by intersecting serotonin-related module genes and bulk-DEGs, which were defined as differentially expressed serotonin-related genes (DE-SRGs). Gene Ontology (GO) annotation and Kyoto Encyclopedia of Genes and Genomes (KEGG) pathway enrichment analysis were performed for the DE-SRGs using the clusterProfiler package (v 4.15.0) [16] (adjusted *p* < 0.05). To clarify the relationships among DE-SRGs, a PPI network was constructed using the Search Tool for the Retrieval of Interacting Genes/Proteins (STRING) database [17]. This approach allowed for the identification of interactions between protein-coding genes with an interaction score > 0.14. The cytoHubba plugin in Cytoscape (v 3.8.2) [18] was employed to identify tightly interactive sub-networks. The top 100 genes ranked by degree were screened to construct the sub-network.

### 2.5. Machine Learning Algorithms

Machine learning techniques were of paramount importance in data mining, especially for model training in classification and prediction. In GSE26155, five machine learning algorithms were applied to evaluate the diagnostic value of the top 100 DE-SRGs. Random seeds were fixed across all machine learning analyses to guarantee result reproducibility. The glmnet package (v 4.1.8) [19] was used to perform Least Absolute Shrinkage and Selection Operator (LASSO) analysis on DE-SRGs to identify LASSO-signature genes, using 10-fold cross-validation with settings family = “binomial”, type.measure = “deviance”, alpha = 1, nfolds = 10; genes were selected based on lambda.min. The randomForest package (v 4.7-1.2) [20] was used to construct the random forest (RF) model, and the importance function was employed to calculate the importance scores. The optimal hyper-parameters ntree = 70 and mtry = 5 were determined by scanning ntree from 10 to 100 (step size = 2) and comparing out-of-bag errors. Gene screening using Support Vector Machine-Recursive Feature Elimination (SVM-RFE) was performed with the caret package (v 6.0.94) [21]. We adopted 5-fold cross-validation (rfeControl (method = “cv”, number = 5), method = “svmRadial”), where feature selection was conducted within each training fold to avoid data leakage. Genes corresponding to the minimum error rate were selected. The Boruta algorithm was implemented using the Boruta package (v 8.0.0) [22] by comparing the importance of each feature among the DE-SRGs with that of randomly generated control features, with pValue = 0.05 and maxRuns = 1000. The xgboost package (v 2.1.1.1) [23] was used for XGBoost analysis. Grid-search hyperparameter tuning yielded final settings: max_depth = 5, eta = 0.1, nround = 100, subsample = 0.7, colsample_bytree = 0.7, gamma = 0.1. Genes with top-ranked Gain values were selected as XGBoost-signature genes. Finally, signature genes identified by all five methods were selected to ensure the stability and reliability of the screening results. To mitigate optimistic bias induced by repeated reuse of the same discovery dataset, a nested cross-validation framework was implemented. The outer layer adopted 5-fold cross-validation to partition samples into outer training and held-out outer test sets. Within each inner training fold, differential expression filtering (Wilcoxon test, *p* < 0.05) was performed first, followed by five machine-learning algorithms; intersecting signature genes from these five algorithms were obtained inside each inner fold. An elastic-net logistic regression model was then built using the intersected features, and predictive performance was evaluated on the corresponding outer held-out test fold. Gene-selection frequency across all five outer folds was calculated to assess the stability of feature candidates.

### 2.6. Identification of Biomarkers and Creation of Nomogram

To validate signature genes’ ability to distinguish samples, the expression levels of signature genes between control and TAA groups were analyzed in GSE26155 and GSE202267. Meanwhile, biomarkers with significantly differential expression and consistent trends between the two groups were identified (Wilcoxon test, *p* < 0.05).

To assess the association between biomarkers and serotonin-pathway activity, a sensitivity analysis was performed based on the original 155-gene SRG set, and Spearman correlation analysis was conducted between the ssGSEA scores of SRGs and biomarker expression levels (|cor| > 0.3, *p* < 0.05).

To further appraise the overall predictive capability of biomarkers for TAA, a nomogram was established using the rms package (v 6.8-1) [24] in GSE26155. Subsequently, the regplot package (v 1.1) was used to plot calibration curves to reflect the predictive performance of the nomogram model. The ggDCA package (v 1.1) [25] was employed to generate decision curve analysis (DCA) curves to evaluate the clinical utility of the nomogram. Receiver operating characteristic (ROC) curves were produced via the pROC package (v 1.18.5) [26] (area under the curve (AUC) value > 0.7). Bootstrap optimism-correction with 500 resamples and 10-fold cross-validation were performed within the training cohort to evaluate internal model stability. Calibration performance was assessed using bootstrap-corrected calibration curves, and the mean absolute error (MAE) was calculated.

### 2.7. Gene Set Enrichment Analysis (GSEA)

In control and TAA samples from GSE26155, the Spearman correlation values between each biomarker and all other genes were calculated using the stats package (v 4.3.3) [27]. Then, the correlation values were ranked in descending order, and GSEA was performed using the clusterProfiler package (v 4.8.2) to further determine the possible biological functions of the biomarkers (adjusted *p* < 0.05, and |NES| > 1). Notably, the reference gene set c2.cp.kegg.v7.4.symbols.gmt was derived from MSigDB.

### 2.8. Immune Infiltration Analysis

The relative infiltration levels of 22 immune cell types [28] from TAA samples and normal controls in the training set GSE26155 were estimated using the CIBERSORT algorithm. Samples with *p* > 0.05 were excluded from subsequent analysis. The Wilcoxon test was used to compare the differences in immune cell infiltration between the two groups (adjusted *p* < 0.05). Spearman correlation analyses were conducted among differentially infiltrated immune cells and between biomarkers and differentially infiltrated immune cells using the psych package (v 2.4.6.26) [29] (|cor| > 0.3, adjusted *p* < 0.05).

### 2.9. Chromosomal, Subcellular Localization, GeneMANIA, and Molecular Regulatory Network

The chromosomal localization of the biomarkers was visualized using the RCircos package (v 1.2.2) [30]. The FASTA format files of the target proteins corresponding to biomarkers were obtained from the National Center for Biotechnology Information (NCBI, https://www.ncbi.nlm.nih.gov/). Subcellular localization prediction was then performed using the CELLO online tool (v 2.5) [31]. The GeneMANIA platform (http://www.genemania.org, accessed on 10 April 2026) [32] was used to integrate additional gene-function association information, facilitating further exploration of the potential biological functions of biomarkers. Next, miRTarBase (v 10.0) [33], miRDB (v 6.0) [34] and TargetScan (v 8.0) [35] were employed to predict microRNAs (miRNAs) that regulate the biomarkers. The miRNA–mRNA regulatory network was constructed using Cytoscape software (v 3.8.2).

### 2.10. Compound Prediction and Molecular Docking

To identify active compounds associated with the biomarkers, the Comparative Toxicogenomics Database (CTD) [36] was used for candidate discovery. The compound with the highest interaction score was selected for molecular docking analysis. The structures of the biomarkers were retrieved from the Protein Data Bank (PDB) [37]. The structures of the predicted compounds were retrieved from the PubChem database [38]. Molecular docking between targets and components was performed using the CB-Dock2 web server [39]. Docking was performed with default parameters: 10 output conformations, box size of 40 × 40 × 40 Å, automatically calculated box center, and Vina Docking Score as the scoring function. A molecular binding energy ≤ −5.0 kcal/mol indicated favorable binding activity.

### 2.11. The scRNA-Seq Data Processing

In all samples from GSE155468, the gene expression matrix was generated using the Read10X function from the Seurat package (v 5.1.0) [40]. A Seurat object was subsequently created with the CreateSeuratObject function. scRNA-seq data were filtered to select high-quality cells, with the following criteria: genes expressed in more than 3 cells, 200 < nCount RNA < 25,000, 200 < nFeature RNA < 5000, and percent.mt < 10%. Data normalization was performed using the LogNormalize function. The FindVariableFeatures function was employed to extract and display the top 2000 highly variable genes (HVGs). Batch-effect correction was performed using the Harmony algorithm, with sample origin (samples) defined as the batch variable, to correct the PCA space prior to dimensionality reduction and clustering. The ScaleData function was used to scale the single-cell expression matrix. Principal component analysis (PCA) was performed using the JackStraw function, and *p*-values were calculated to identify statistically significant principal components (PCs) (*p* < 0.05). Uniform manifold approximation and projection (UMAP) was applied for dimensionality reduction and determination of clusters via the FindNeighbors and FindClusters functions (resolution = 0.2). Cell clusters were identified using the FindAllMarkers function to obtain specifically highly expressed genes with the following parameters: min.pct = 0.25, logfc.threshold = 0.25, and test.use = wilcoxon. Cells were annotated as different types based on marker genes using the literature [41]. Subsequently, the proportion of each cell type was calculated per donor sample, and the Wilcoxon test was performed to compare differences between groups (*p* < 0.05). Next, pseudo-bulk expression analysis was conducted for biomarkers. The counts were aggregated for each donor-cell-type combination to compute log_2_Counts Per Million (CPM) values, and group-wise comparisons were then performed at the donor level. Distribution and expression levels of serotonin-related genes across different cell types were visualized. Functional enrichment analysis was performed on annotated cells using the ReactomeGSA package (v 1.14.0) [42].

### 2.12. Single-Cell Gene Knockout Simulation

To elucidate the effects of biomarker knockout on the function of key cells, the scTenifoldKnk package (v 1.0.2) [43] was employed to construct gene regulatory networks (GRNs). Genes with virtual knockout perturbations were selected under the threshold of FDR-*p* value < 0.01 and |Z| > 2. To explore the potential functions and signaling pathways of the knockout-perturbed genes, GO and KEGG enrichment analyses were performed using the clusterProfiler package (v 4.8.3) (*p* < 0.05).

### 2.13. Characterization of Key Cells

In the single-cell dataset GSE155468, the FindMarkers function was used to identify single-cell-DEGs in key cells (| log2 FC| > 0.5 and *p* < 0.05). Subsequently, GO and KEGG enrichment analyses were performed on the single-cell-DEGs. The CellChat package (v 2.2.0) [44] was utilized to analyze the communication among the annotated cells. The ligand–receptor networks and the signals from each annotated cell were visualized. The monocle2 package (v 2.30.1) [45] was utilized for pseudotime analysis to examine the differentiation status of key cells and to assess the changes in biomarker expression levels. Cell cycle phases (G1, S, or G2/M) of key cells were assigned using the CellCycleScoring function. Moreover, based on the SCENIC algorithm [46], transcription factors (TFs) in key cell subtypes were identified.

Finally, the key cellular compartment was reannotated into distinct subtypes, and the distributions of biomarkers and SRG scores across these subtypes were analyzed.

### 2.14. Reverse Transcription Quantitative PCR (RT-qPCR)

The 5 TAA tissue specimens and 5 control tissues were collected at Zhongshan Hospital Affiliated with Fudan University. Approval was granted by the Ethics Committee of Zhongshan Hospital, Fudan University (Approval No. B2025-413R). Total RNA was extracted, and complementary DNA synthesis was performed using the cDNA synthesis kit (Servicebio, Wuhan, China) according to the manufacturer’s instructions. Quantitative real-time PCR was performed in triplicate using SYBR Green Master Mix. GAPDH was used as a housekeeping control, and the relative expression of biomarkers was calculated using the 2^−∆∆Ct^ method. The sequences of the primers used for quantitative real-time PCR are listed in Table S2.

### 2.15. Statistical Analysis

All data were processed using R language software (v 4.3.3) [42]. Comparisons were analyzed through the Wilcoxon test or t-test, and a *p* < 0.05 was considered statistically significant. Differential expression analysis, enrichment analysis, and immune infiltration analysis all utilized *p*-values adjusted for Benjamini–Hochberg correction.

## 3. Results

### 3.1. Identification of Bulk-DEGs and Serotonin-Related Module Genes

**Figure 1:**
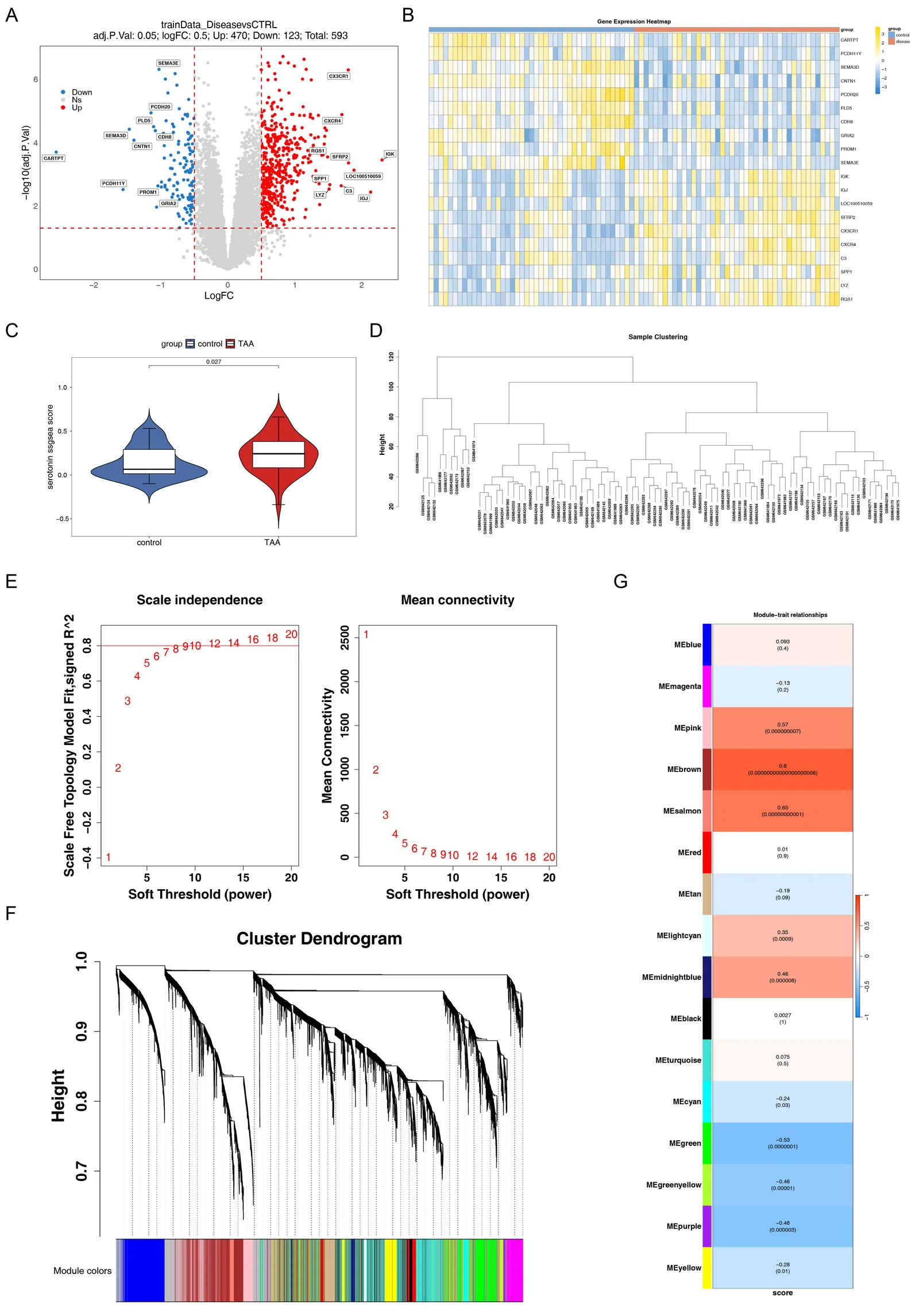
Identification of bulk-DEGs and serotonin-related module genes. (**A**) Volcano plot showing bulk-differentially expressed genes (bulk-DEGs) between TAA and control samples. (**B**) Heatmap showing the expression patterns of representative bulk-DEGs. (**C**) Boxplot showing differences in serotonin-related gene set scores between TAA and control samples. (**D**) Sample clustering dendrogram for WGCNA. (**E**) Analysis of soft-thresholding power. (**F**) Gene clustering dendrogram with module assignment. (**G**) Module–trait correlation heatmap identifying serotonin-related modules. DEGs, differentially expressed genes; TAA, thoracic aortic aneurysm; WGCNA, weighted gene co-expression network analysis.

In GSE26155, 593 bulk-DEGs (Table S3) were determined between control and TAA samples (Figure 1A,B). Among them, 470 genes were upregulated and 123 genes were downregulated in the TAA samples. ssGSEA analysis revealed that the score of the SRG set was significantly elevated in the TAA group (*p* < 0.05), suggesting aberrant activation of the serotonin signaling pathway during the development and progression of TAA (Figure 1C). No samples exhibited obvious outlier deviation (Figure 1D). According to the scale-free network distribution criterion, a soft threshold power of 10 was selected as the optimal value, and a total of 16 gene co-expression modules with distinct clustering characteristics were identified (Figure 1E,F). Genes in the MEbrown (cor = 0.8, *p* < 0.0001) and MEgreen modules (cor = −0.53, *p* < 0.0001) were incorporated to expand the SRG set, yielding a final set of 1731 serotonin-related module genes (Table S4; Figure 1G).

**Figure 2:**
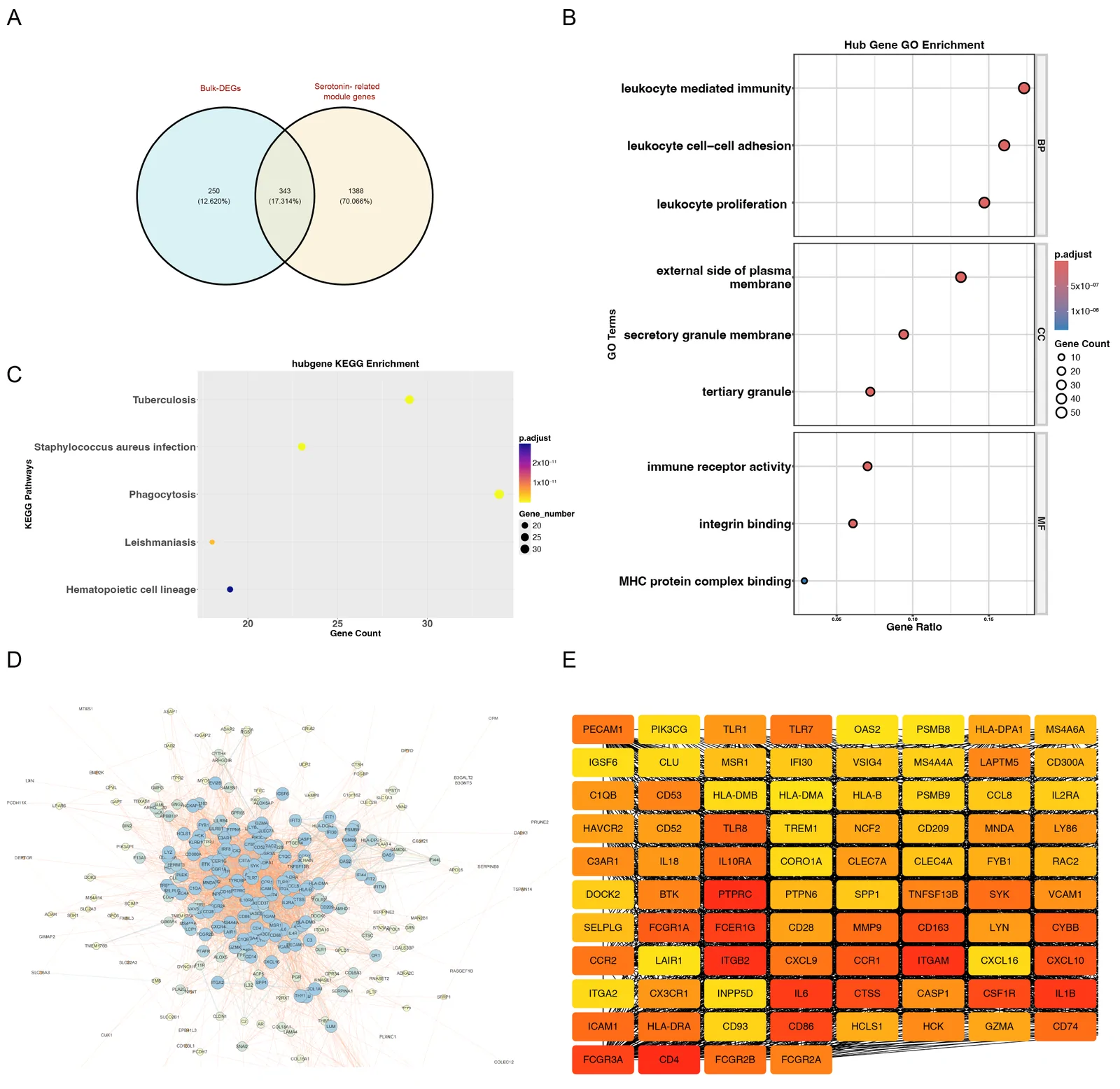
Functional assessment of DE-SRGs. (**A**) Venn diagram showing the intersection between bulk-DEGs and serotonin-related module genes. (**B**) Gene Ontology (GO) enrichment analysis of DE-SRGs. (**C**) Kyoto Encyclopedia of Genes and Genomes (KEGG) pathway enrichment analysis of DE-SRGs. (**D**) Protein–protein interaction (PPI) network of DE-SRGs. (**E**) cytoHubba subnetwork showing the top 100 DE-SRGs ranked by degree. DE-SRGs, differentially expressed serotonin-related genes, node colors from yellow to red indicate increasing degree values, with red representing genes with higher network connectivity.

Functional Enrichment Analysis of DE-SRGs. A total of 343 DE-SRGs (Table S5) were obtained by intersecting 593 bulk-DEGs and 1731 serotonin-related module genes (Figure 2A). GO and KEGG analyses were performed for the DE-SRGs to elucidate the molecular biological processes. A total of 961 biological process (BP) terms were enriched including leukocyte mediated immunity and leukocyte cell–cell adhesion, 72 molecular function (MF) terms, including immune receptor activity and integrin binding; and 68 cellular component (CC) terms, including external side of plasma membrane and secretory granule membrane (adjusted *p* < 0.05, Table S6, Figure 2B). DE-SRGs were enriched in 66 KEGG pathways, such as phagosome and hematopoietic cell lineage (adjusted *p* < 0.05, Table S7, Figure 2C). The protein interactions among the DE-SRGs were investigated. A subnetwork was generated from the top 100 genes (Table S8) ranked by degree, in which CD4, PTPRC, ITGB2, TYROBP, and ITGAM showed extensive interactions with other proteins (Figure 2E). Since ties existed in the degree values at the 100-th rank, a total of 104 genes were included in practice. These enrichment results indicated that the DE-SRGs might play crucial roles in regulating inflammatory responses, thereby providing potential mechanistic insights into the pathogenesis and progression of TAA.

### 3.2. Identification and Validation of CD300A and CLU as Biomarkers

**Figure 3:**
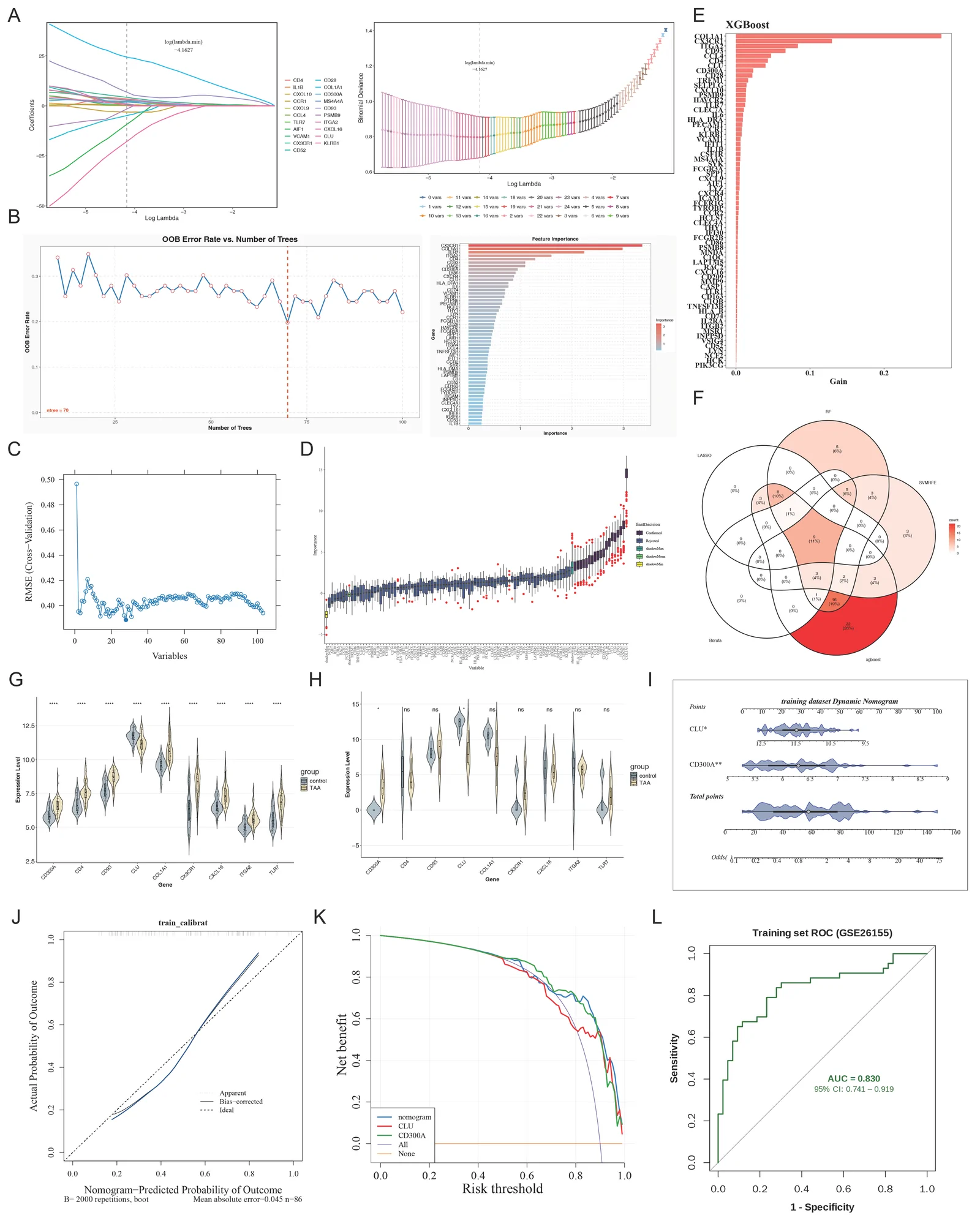
Identification and validation of CD300A and CLU as biomarkers. (**A**) Signature genes identified by LASSO regression. (**B**) Gene importance ranking based on random forest analysis. (**C**) Feature genes selected using the SVM-RFE algorithm. (**D**) Boruta analysis showing genes classified as important. (**E**) XGBoost-based feature importance ranking. (**F**) Venn diagram showing overlapping signature genes identified by the five machine learning algorithms. (**G**,**H**) Violin plots showing the expression levels of signature genes in GSE26155 and GSE202267. (**I**) Nomogram based on CD300A and CLU. (**J**) Calibration curve of the nomogram. (**K**) Decision curve analysis (DCA). (**L**) Receiver operating characteristic (ROC) curves. ns, not significant; * *p* < 0.05; ** *p* < 0.01; **** *p* < 0.0001.

Machine learning algorithms enable the identification of signature genes associated with serotonin in TAA. The optimal model was constructed when the model error reached its minimum, with a log(λmin) of −4.1627. A total of 21 LASSO-signature genes were retained, including CD4, IL1B, CXCL10, CCR1, CXCL9, CCL4, TLR7, AIF1, VCAM1, CX3CR1, CD52, CD28, COL1A1, CD300A, MS4A4A, CD93, PSMB9, ITGA2, CXCL16, CLU, and KLRB1 (Figure 3A). For the RF model, the top 53 RF-signature genes with a relative importance score greater than 0.25 were identified (Figure 3B). By selecting the most relevant features, the SVM-RFE algorithm identified 28 signature genes corresponding to the lowest model error rate: COL1A1, CX3CR1, CD93, TLR7, CD4, OAS2, ITGA2, CD300A, CXCL16, IRF8, LY86, CXCR4, CD74, CLU, HLA-DPA1, LAIR1, FCGR3A, SYK, HCLS1, ITGA4, CD53, PTPRC, LCP2, CSF1R, MNDA, PTPN6, IL10RA, and ITGB2 (Figure 3C). The Boruta algorithm was applied to extract genes defined as “important”, and 19 Boruta-signature genes were retained (Figure 3D). For the XGBoost model, 68 XGBoost-signature genes were retained based on feature importance evaluated by calculating the gain of each feature in the model (Figure 3E). The five machine learning methods were utilized to intersect the signature genes of TAA, resulting in 9 signature genes, namely CD4, TLR7, CX3CR1, COL1A1, CD300A, CD93, CXCL16, ITGA2, and CLU (Figure 3F). The expression trends of the signature genes were validated in the control and TAA groups from the GSE26155 and GSE202267 datasets. The results revealed that CD300A was significantly upregulated, while CLU was significantly downregulated in the TAA group (*p* < 0.05, Figure 3G,H). Consequently, CD300A and CLU were designated as biomarkers. To assess potential optimistic bias from repeated dataset reuse, nested 5-fold cross-validation was performed. The outer-fold average AUC reached 0.902 (SD = 0.06, 95%CI: 0.849–0.955) (Figure S1A), which was comparable to the original analysis and indicated stable predictive performance. Gene-selection-frequency analysis across outer folds showed that CD4, CD93, COL1A1, CX3CR1, and TLR7 were selected in all five folds (100% frequency), while CD300A and CXCL16 were selected in four out of five folds (80% frequency) (Figure S1B). The high selection frequency supported CD300A and CLU as reliable biomarker candidates. Spearman correlation analyses showed that CD300A was positively correlated with the original-SRG-derived serotonin pathway score (cor = 0.651, *p* < 0.001), whereas CLU exhibited a significant negative correlation (cor = −0.568, *p* < 0.001) (Figure S1C). Notably, CD300A and CLU were not members of the original curated SRG list, indicating they likely act as downstream effectors of serotonin-driven transcriptional responses in TAA. A TAA diagnostic nomogram model was developed according to the biomarkers (CD300A and CLU) from GSE26155. The higher the total points, the greater the probability of suffering from TAA (Figure 3I). There was no significant difference between the predicted values and the actual observed values, indicating favorable calibration of the model (Figure 3J). DCA demonstrated that the nomogram curve exceeded the curves illustrating no intervention, all interventions, and all single biomarkers, implying that the nomogram had favorable clinical utility (Figure 3K). An AUC value of 0.830 (95%CI:0.741–0.919) indicated that the nomogram model had great reference value for the diagnosis of TAA (Figure 3L). After bootstrap optimism-correction with 500 resamples, the optimism-corrected AUC was 0.824 (Figure S1D). Ten-fold cross-validation yielded a mean AUC of 0.812 (SD = 0.168) (Figure S1E). The bootstrap-corrected calibration curve showed good agreement between predicted and observed probabilities with a mean absolute error (MAE) of 0.044 (Figure S1F). DCA demonstrated that the nomogram achieved higher net benefit than the treat-all or treat-none strategies across a broad threshold range (Figure S1G). Given the extremely small sample size of the independent dataset GSE202267 (4 TAA, 4 controls), formal external validation could not be reliably performed. Collectively, these internal evaluations support this as an exploratory diagnostic model for TAA.

### 3.3. GSEA of the Biomarkers in TAA

**Figure 4:**
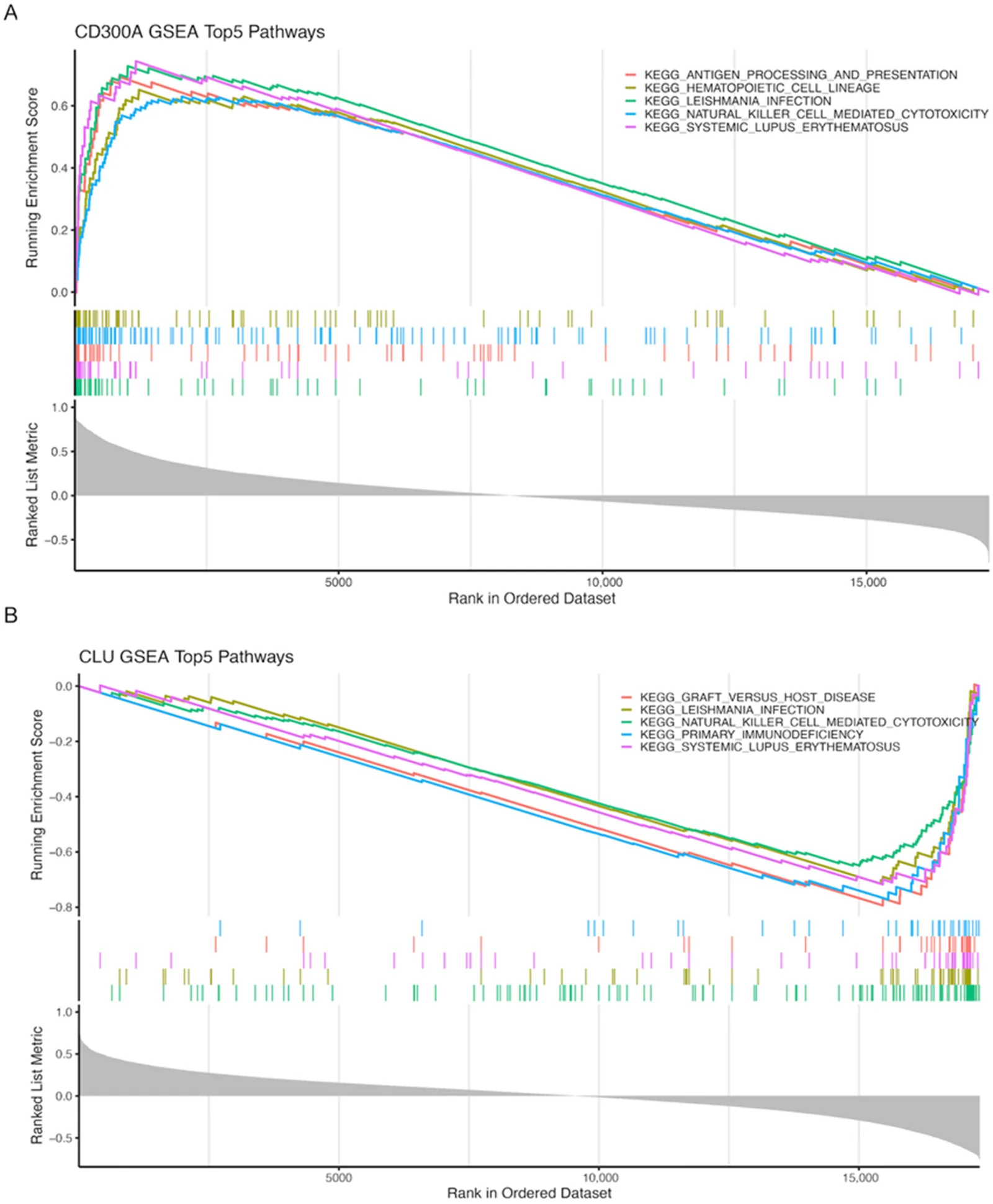
Biological mechanisms associated with biomarkers in TAA. Gene set enrichment analysis (GSEA) showing pathways significantly associated with CD300A (**A**) and CLU (**B**) in TAA. TAA, thoracic aortic aneurysm. The gray area represents the ranked list metric across the ordered genes.

GSEA is particularly valuable for elucidating comprehensive and coherent changes in biological processes. In TAA, GSEA for CD300A was closely related to 57 functional pathways, such as natural killer cell mediated cytotoxicity (adjusted *p* < 0.05, Table S9, (Figure 4A)). CLU was significantly enriched in 55 functional pathways, including antigen processing and presentation, hematopoietic cell lineage, and natural killer cell mediated cytotoxicity (adjusted *p* < 0.05, Table S10, Figure 4B). CD300A and CLU might play important roles in the pathological process of TAA by extensively participating in immune recognition, immune cell activation, and immune regulation imbalance.

### 3.4. Immune Landscape Associated with Biomarkers in TAA

**Figure 5:**
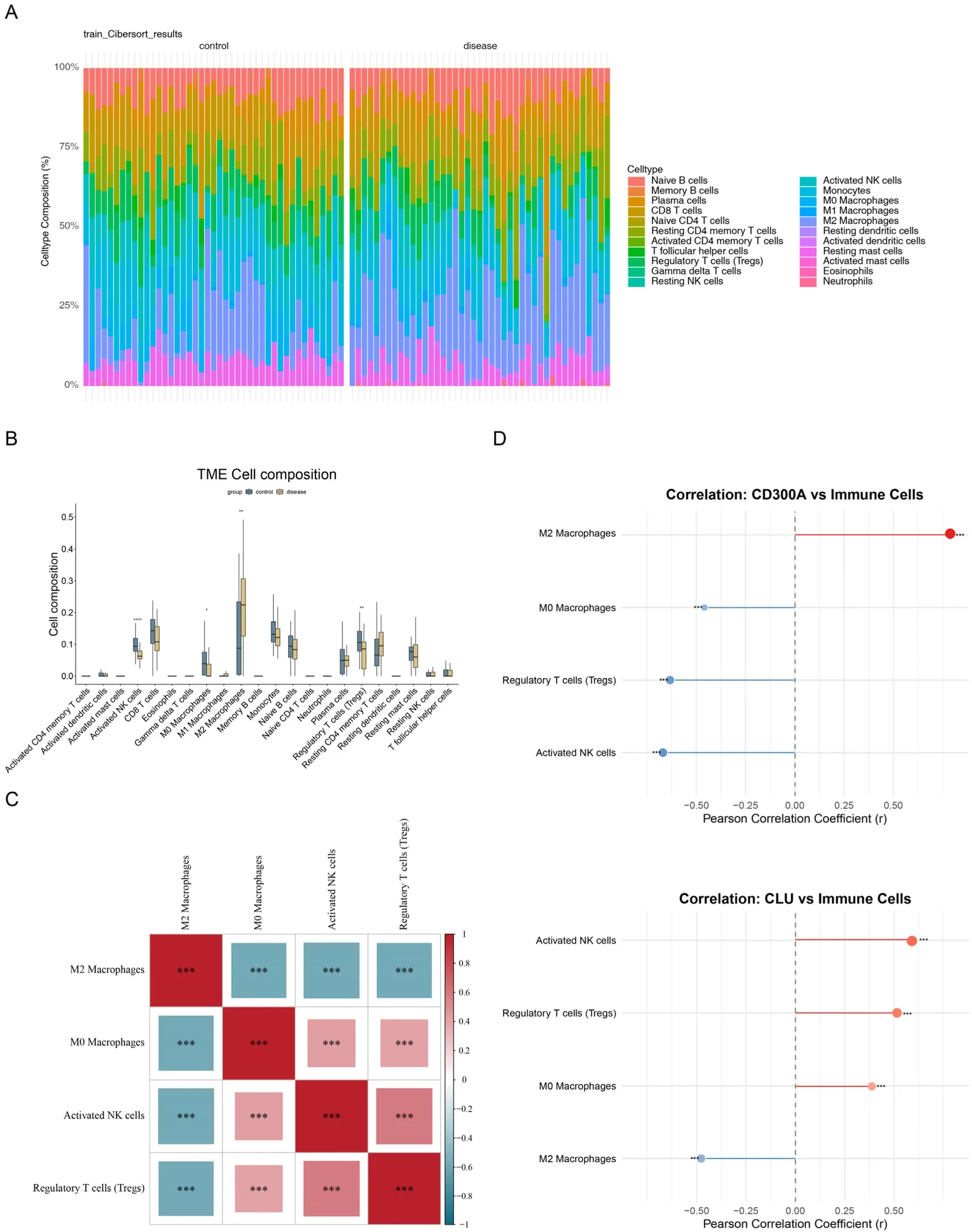
Immune infiltration in TAA. (**A**) Heatmap showing the infiltration levels of 22 immune cell types. (**B**) Boxplot comparing immune cell infiltration between TAA and control samples. (**C**) Correlation analysis among differentially infiltrated immune cells, red and blue indicate positive and negative correlations, respectively. (**D**) Correlation analysis between biomarkers and differentially infiltrated immune cells. TAA, thoracic aortic aneurysm. * adjusted *p* < 0.05; ** adjusted *p* < 0.01; *** adjusted *p* < 0.001; **** adjusted *p* < 0.0001.

To conduct a more in-depth exploration of the immune microenvironment in TAA, immune infiltration analysis was carried out using data from the GSE26155 dataset (Figure 5A). Four types of immune cells were found to have different distributions: the infiltration levels of M2 macrophages were markedly upregulated in TAA samples compared with control samples, while those of regulatory T cells (Tregs), M0 macrophages, and activated natural killer (NK) cells were significantly downregulated in TAA samples (adjusted *p* < 0.05, Figure 5B). Tregs showed a strong positive correlation with activated NK cells (cor = 0.58, adjusted *p* < 0.001). M2 macrophages showed a strong negative correlation with activated NK cells (cor = −0.58, adjusted *p* < 0.001) (Table S11, Figure 5C). CD300A expression was significantly positively correlated with the infiltration proportions of M2 macrophages (cor = 0.79, adjusted *p* < 0.001), but significantly negatively correlated with those of M0 macrophages (cor = −0.46, adjusted *p* < 0.001), Tregs (cor = −0.63, adjusted *p* < 0.001), and activated NK cells (cor = −0.67, adjusted *p* < 0.001). In contrast, CLU expression exhibited an entirely opposite correlation pattern (Figure 5D). These results suggested that the expression levels of these biomarkers are closely associated with specific immune cell infiltration patterns in TAA.

### 3.5. Comprehensive Regulatory Analysis of Biomarkers

**Figure 6:**
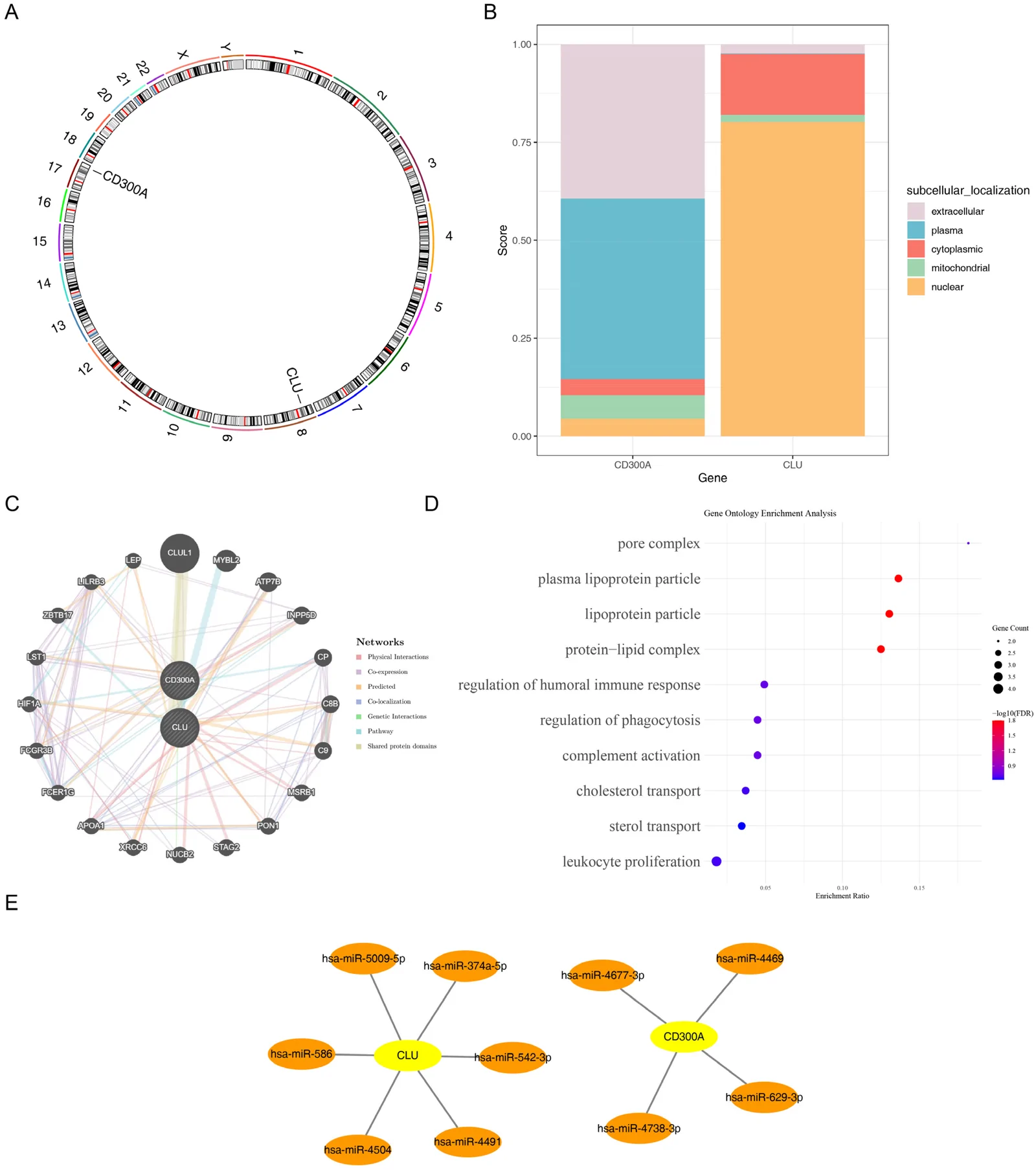
Comprehensive regulatory analysis of biomarkers. (**A**) Chromosomal localization of CD300A and CLU, numbers indicate chromosomes and colors distinguish chromosomes. (**B**) Predicted subcellular localization of the biomarkers. (**C**) Gene–gene interaction network of the biomarkers generated using GeneMANIA. (**D**) Shared functional pathways identified by GeneMANIA. (**E**) miRNA–mRNA regulatory network. Orange nodes represent miRNAs, and yellow nodes represent mRNAs.

Chromosomal localization revealed that CLU was located on chromosome 8, while CD300A was mapped to chromosome 17 (Figure 6A). Subcellular localization prediction indicated that CD300A was mainly distributed in the extracellular region and plasma membrane, suggesting that it may act as a membrane receptor or secreted protein, mediating immune recognition and signal transduction on the cell surface or in the extracellular microenvironment. CLU showed an extremely high probability of nuclear localization, implying that it was potentially involved in diverse biological processes including secretory pathways, intranuclear regulation, and stress responses (Figure 6B). Based on GeneMANIA interaction network analysis, CD300A and CLU were associated with a tightly interacting network consisting of 20 genes, including CLU, MYBL2, ATP7B, ZBTB17, CP, PON1, C9, MSRB1, INPP5D, LEP, LEF1, NUCB2, among others (Figure 6C). These genes were involved in shared signaling pathways, such as pore complex, plasma lipoprotein particle, and lipoprotein particle (Figure 6D). Considering the pivotal roles of the two biomarkers, studies were carried out to predict miRNAs to delineate a multifaceted regulatory landscape. Six miRNAs (such as hsa-miR-586 and hsa-miR-542-3p) were predicted to target and regulate CLU, whereas four miRNAs (such as hsa-miR-629-3p and hsa-miR-4469) were identified to target CD300A (Figure 6E). This network analysis strongly supported the elucidation of the regulatory mechanisms underlying TAA.

### 3.6. Docking Results of Key Compounds and Biomarkers

**Figure 7:**
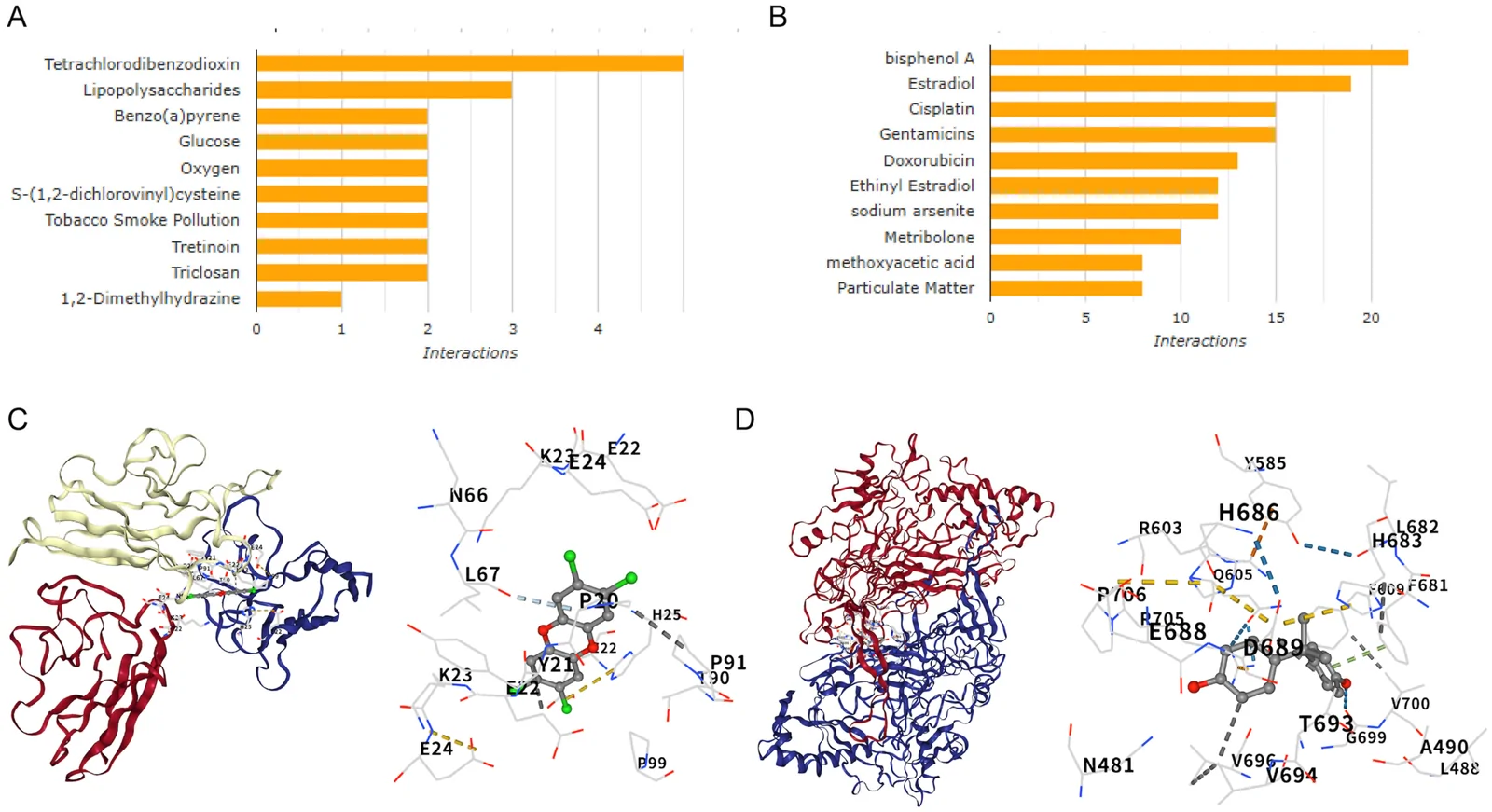
Molecular docking between compounds and biomarkers in TAA. (**A**,**B**) Histograms showing compound–biomarker interaction scores for CD300A (**A**) and CLU (**B**). (**C**) Molecular docking of tetrachlorodibenzodioxin with CD300A. (**D**) Molecular docking of bisphenol A with CLU. TAA, thoracic aortic aneurysm.

Among the interacting compounds of CD300A, Tetrachlorodibenzodioxin showed the strongest interaction (Figure 7A,B). For CLU, bisphenol A exhibited the most abundant interactions. Molecular docking results revealed that Tetrachlorodibenzodioxin bound to the protein encoded by CD300A with a binding energy of −6.6 kcal/mol, interacting via residues including P20 and Y21 (Figure 7C). Bisphenol A bound to the protein encoded by CLU with a binding energy of −7.9 kcal/mol, interacting via residues including D689, E688, and T693 (Figure 7D). These docking outputs indicate computationally stable binding interfaces between small-molecule ligands and target proteins. However, such in-silico results do not prove physiological or therapeutic relevance for TAA.

### 3.7. Identification of Key Cells in scRNA-Seq Dataset

**Figure 8:**
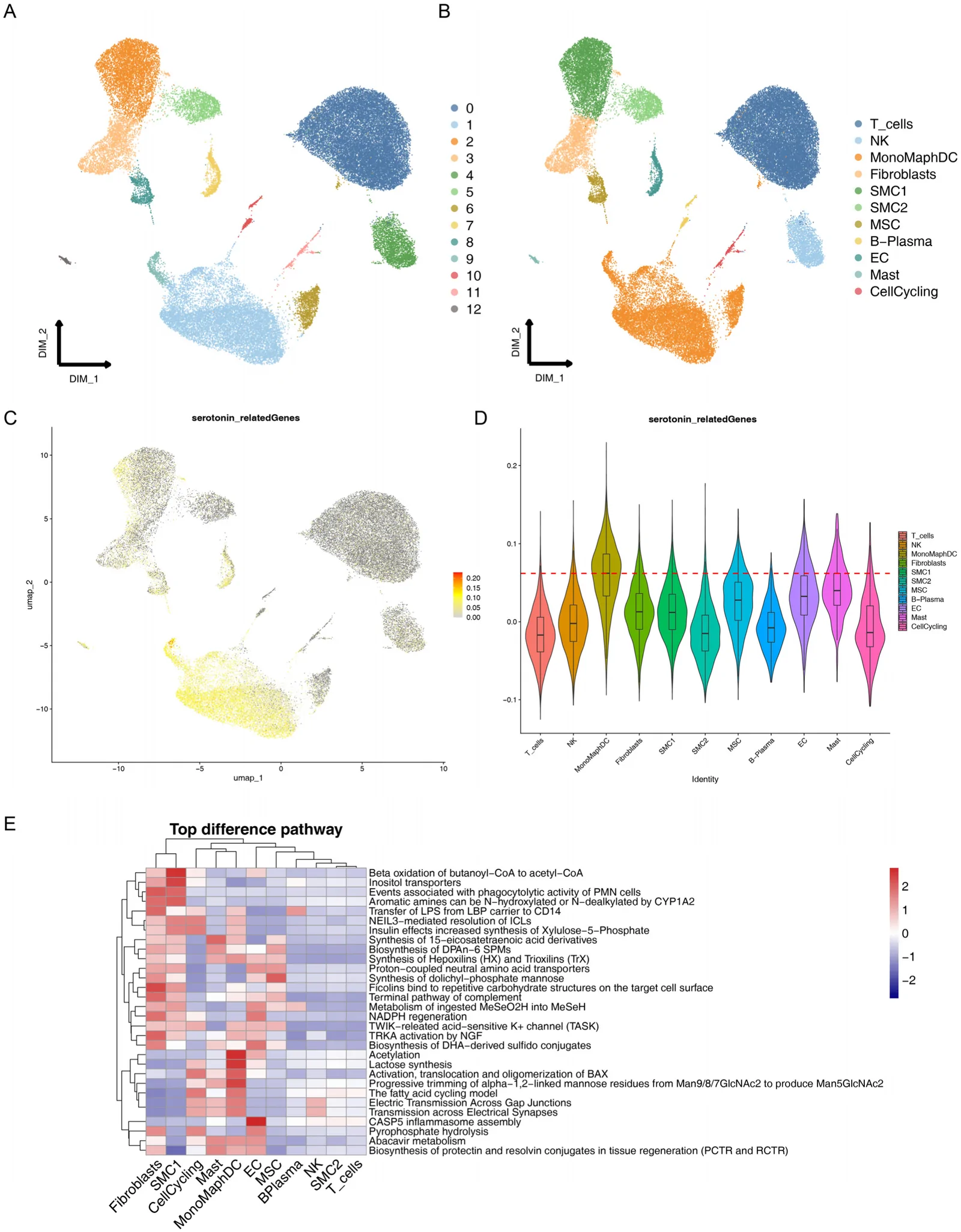
Identification of key cells. (**A**) Uniform Manifold Approximation and Projection (UMAP) plot showing single-cell clusters, numbers 0–12 indicate single-cell cluster IDs. (**B**) UMAP plot showing annotated cell types. (**C**) UMAP plot showing serotonin-related gene set scores across single cells. (**D**) Violin plots showing serotonin-related gene set scores across different cell types. (**E**) Heatmap showing pathway enrichment scores in MonoMac/DC based on GSVA. MonoMac/DC, monocytes/macrophages/dendritic cells; GSVA, gene set variation analysis, the red dashed line indicates the predefined reference cutoff for the serotonin-related gene set score.

Initially, after integrating and filtering the original data of GSE155468 for TAA, the data contained 48,128 cells and 22,772 genes before quality control (QC) and 47,517 cells and 22,772 genes after QC. The top 2000 HVGs and top 20 PCs were identified (Figure S2). Then, 13 distinct cell clusters were identified via UMAP clustering analysis (Figure 8A) and 11 cell clusters were annotated (Figure 8B). The bubble plot was employed to display marker gene expressions in different annotated cells. Notably, 11 specific cell types were identified, including T cells (CD3D, CD3E, CD3G), NK (NKG7, GNLY, KLRD1), Monocytes/macrophages/dendritic cells (MonoMac/DC, LYZ, CD14, FCGR3A), fibroblasts (COL1A1, DCN, LUM), SMC (smooth muscle cells; ACTA2, MYH11, TAGLN), MSC (mesenchymal stem cells; MT1M, MT2A, CRISPLD2), B cells (CD79A, MS4A1, CD19), plasma cells (MZB1, SDC1, JCHAIN), Endo (endothelial cells; PECAM1, VWF, CDH5), mast cells (CPA3, TPSAB1, KIT), and cycling cells (MKI67, PCNA, TOP2A). Each sample in the TAA group contained approximately 3024–6967 cells, whereas each control sample contained roughly 1185–3906 cells (Table S12). The TAA group exhibited higher proportions of T cells and MonoMac/DC, along with a relatively lower proportion of SMC1. Pseudo-bulk expression analysis revealed that the expression trends of CD300A across cell types, including MonoMac/DC and T cells were consistent with those observed in bulk data. Similarly, CLU showed bulk-consistent expression trends in fibroblasts and B-plasma cells, although none of these comparisons reached statistical significance (Figure S3). Consistent expression patterns across individual donors were observed, and no major findings were driven by outlier subjects. Notably, the high expression of the serotonin-related gene set was enriched in MonoMac/DC (Figure 8C,D). This cell type was highly enriched in acetylation, lactose synthesis, activation, translocation and oligomerization of BAX, electric transmission across gap junctions, and transmission across electrical synapses (Figure 8E). Therefore, MonoMac/DC were defined as the key cell type in the scRNA-seq dataset of TAA.

### 3.8. Results of Virtual Knockout of Biomarkers in TAA

**Figure 9:**
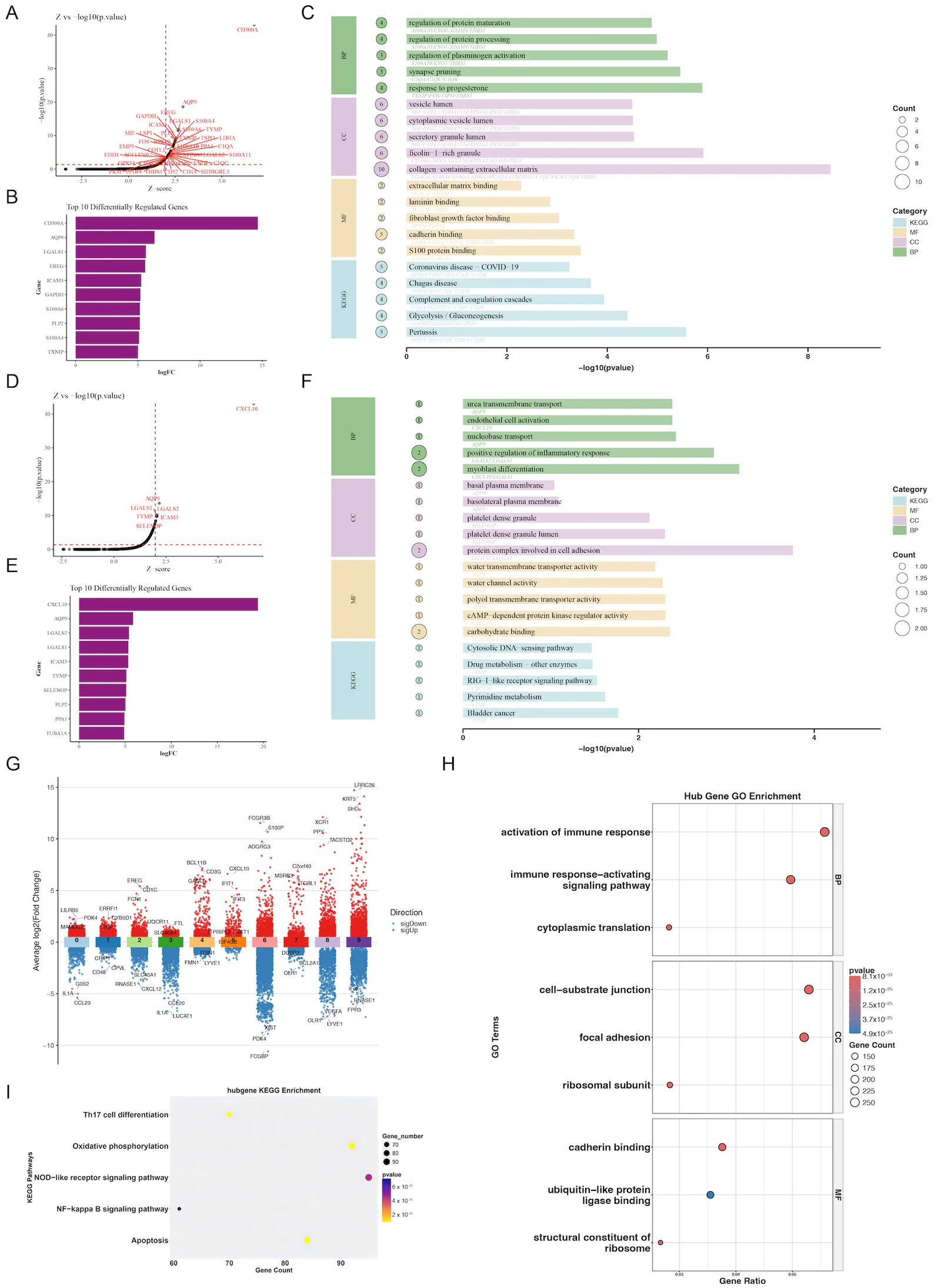
Virtual knockout analysis of biomarkers in TAA. (**A**,**B**) Perturbed genes after virtual knockout of CD300A in MonoMac/DC. (**C**) GO and KEGG enrichment analyses of CD300A-perturbed genes. (**D**,**E**) Perturbed genes after virtual knockout of CLU in MonoMac/DC. Blue dashed line: Z-score cutoff; red dashed line: significance threshold. (**F**) GO and KEGG enrichment analyses of CLU-perturbed genes. (**G**) Differentially expressed genes in MonoMac/DC. (**H**,**I**) GO and KEGG enrichment analyses of differentially expressed genes in MonoMac/DC. TAA, thoracic aortic aneurysm; MonoMac/DC, monocytes/macrophages/dendritic cells.

To elucidate the cellular mechanisms underlying the functions of biomarkers, virtual knockout experiments were performed. For CD300A in MonoMac/DC, 39 perturbed genes, including AQP9, LGALS1, EREG and ICAM3, were identified, all of which showed strong expression changes (Table S12, Figure 9A,B). These genes played central roles in the regulation of protein maturation, vesicle lumen, and S100 protein binding, and were highly enriched in complement and coagulation cascades as well as glycolysis/gluconeogenesis (Tables S13 and S14, Figure 9C). For CLU, 7 perturbed genes were detected, including CXCL10, AQP9, LGALS2, and ICAM3 (Table S15, Figure 9D,E), which played central roles in myoblast differentiation, protein complexes involved in cell adhesion and carbohydrate binding, RIG- I-like receptor signaling pathways, drug metabolism/other enzymes cytosolic DNA-sensing pathways, and were highly enriched in pyrimidine metabolism (Tables S16 and S17, Figure 9F). A total of 14,408 differential genes of MonoMac/DC were identified using the FindMarkers function (Table S18, Figure 9G). GO enrichment analysis showed that these genes were mainly enriched in the activation of immune response, focal adhesion and cadherin binding. KEGG enrichment analysis indicated that the genes were mainly enriched in oxidative phosphorylation, apoptosis, Th17 cell differentiation, NOD-like receptor signaling pathway and NF-κB signaling pathway (Tables S19–S21, Figure 9H,I). Collectively, these in-silico virtual-knockout and differential-gene-profiling results suggest potential regulatory roles of CD300A and CLU in immune activation, inflammatory signaling, and metabolic rewiring within MonoMac/DC. Virtual-knockout simulation represents computational prediction rather than experimental gene perturbation, and functional validation is required to confirm these regulatory effects.

### 3.9. Characterization of MonoMac/DC in TAA

**Figure 10:**
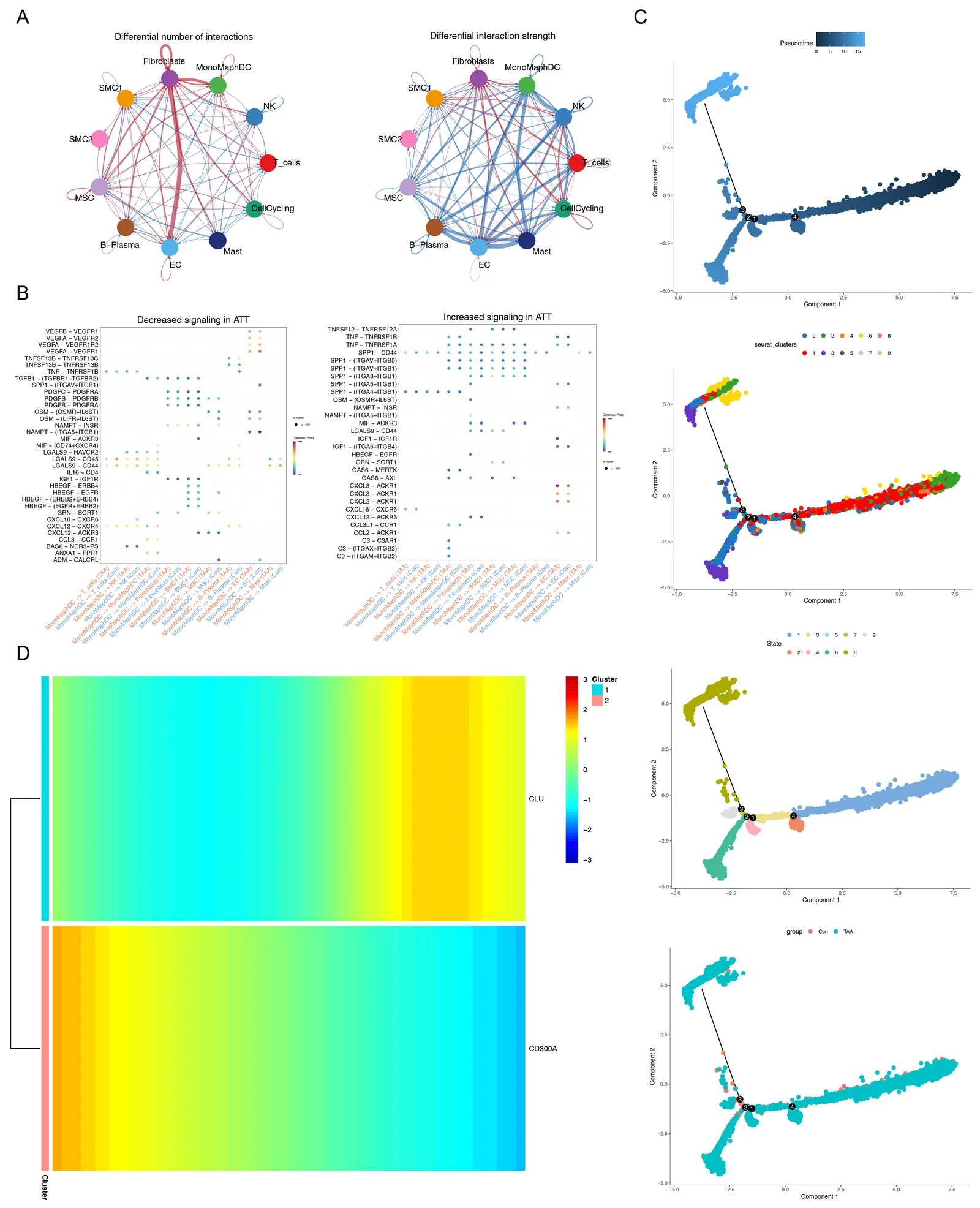
Cell communication and pseudotime analysis. (**A**) Cell–cell communication weight network among annotated cell types. (**B**) Bubble plot showing ligand–receptor interactions between MonoMac/DC and other cell types. (**C**) Pseudotime trajectory analysis of MonoMac/DC. (**D**) Expression trends of CD300A and CLU along the pseudotime trajectory. MonoMac/DC, monocytes/macrophages/dendritic cells.

**Figure 11:**
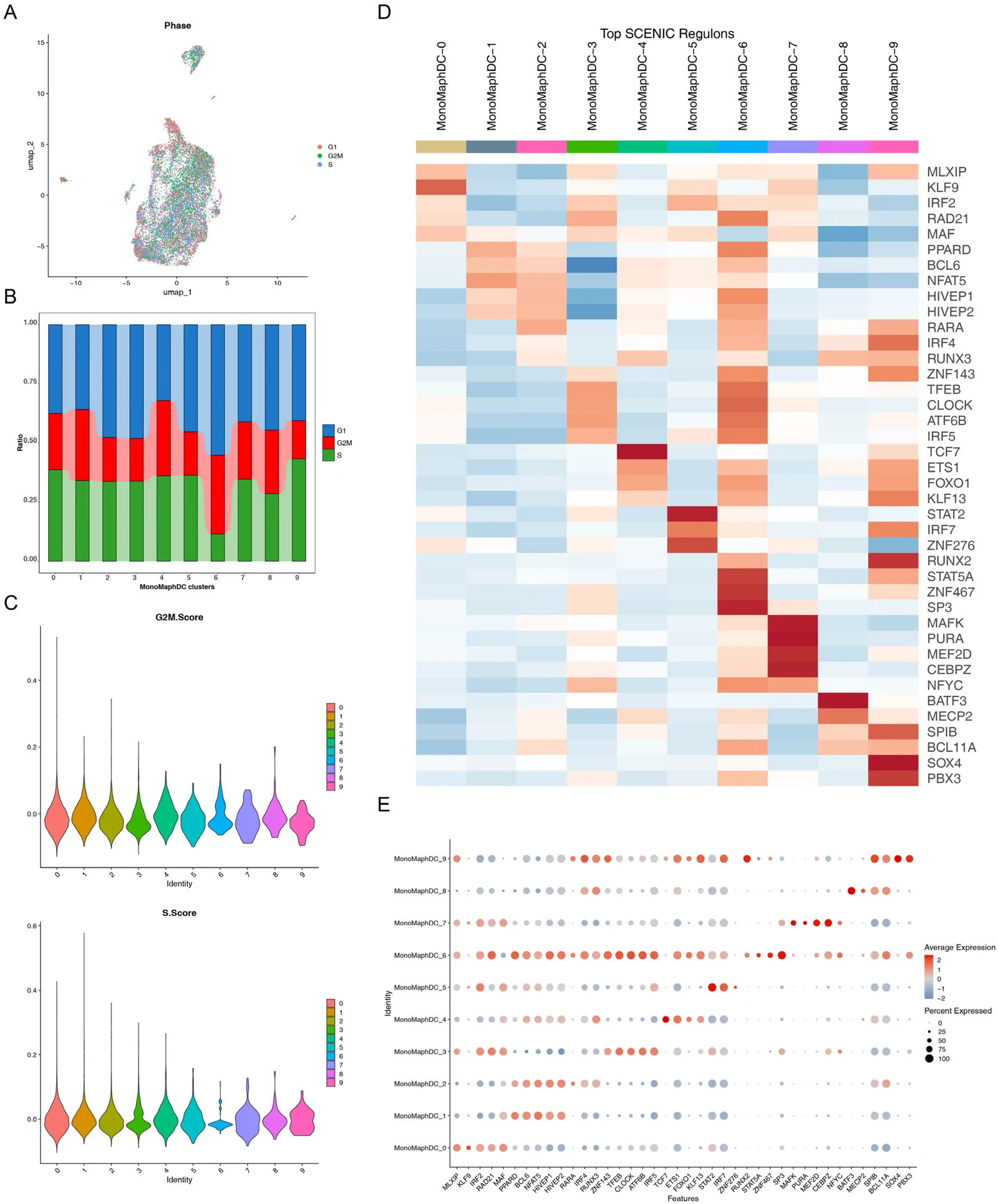
Cell cycle and transcriptional regulation of MonoMac/DC in TAA. (**A**) UMAP plot showing MonoMac/DC colored by cell cycle phase. (**B**,**C**) Expression levels of representative cell cycle-related genes in MonoMac/DC subsets. (**D**) Heatmap showing area under the curve (AUC) scores of transcription factor regulatory activity estimated by SCENIC. (**E**) Dot plot showing the average expression levels of representative transcription factor genes. MonoMac/DC, monocytes/macrophages/dendritic cells; TAA, thoracic aortic aneurysm.

Most intercellular communications were enhanced in the TAA disease group. Interactions between MonoMac/DC and fibroblasts were increased, whereas those with endothelial cells were decreased (Figure 10A). Ligand–receptor pairs significantly upregulated in the disease group mainly included the TNF superfamily (such as TNFSF12–TNFRSF12A and TNF–TNFRSF1A/B), the SPP1– integrin axis (such as SPP1–CD44 and SPP1–integrin complexes), chemokine axes (such as CXCL12–ACKR3 and CCL2–ACKR1), and growth factor signaling (such as IGF1–IGF1R and GAS6–AXL/MERTK) (Figure 10B). These results indicated extensive reprogramming of intercellular communication within the thoracic aortic aneurysm microenvironment, characterized by widespread activation of pro-inflammatory, pro-fibrotic, and growth factor-related pathways. Given the transcriptional heterogeneity of cells in TAA patients, pseudotime analysis was performed on MonoMac/DC subsets, which were divided into 9 stages. State 1 represented the early differentiation state, whereas State 8 corresponded to the late differentiation state (Figure 10C). CD300A expression was highly enriched at the initial stage of the pseudotime trajectory, while CLU was mainly concentrated at the terminal stage (Figure 10D). Their temporal separation along the trajectory provided important sequential molecular clues for generating hypotheses regarding cell-state transitions potentially linked to TAA pathogenesis. It should be noted that pseudotime trajectory inference is a computational prediction and cannot directly prove causal disease progression. Each cell type contained a subset of cells in G1, G2M, and S phases, indicating that partial cells of each type were undergoing cell cycle progression, suggesting enhanced cellular activation (Figure 11A–C). Based on the results of SCENIC transcriptional regulatory network analysis, a series of specifically activated TFs were identified in multiple subsets of MonoMac/DC cells (Figure 11D,E). Members of the interferon regulatory factor family (IRF4, IRF5, IRF7) and the signal transducer and activator of transcription family (STAT2, STAT5A) were coordinately enriched in several late-stage subsets, suggesting that these TFs may collectively contribute to the differentiation and functional specialization of MonoMac/DC cells toward pro-inflammatory or immunoregulatory phenotypes.

The results showed that the 10 MonoMac/DC cell clusters were annotated into 10 cell subtypes, including M2_macrophage (MRC1, LYVE1, STAB1, MAF, MERTK), Inflammatory_macrophage (TNF, IL1A, CCL3, CCL4, NLRP3), EREG+_macrophage (EREG, AREG, IL1B, LYZ, FCGR2B), FTL+_monocyte (FTL, UQCR11, ATP5F1E, RNASE1), CD3E+_monocyte (CD3D, CD3E, CD3G, TRAC, TRBC2), ISG+_monocyte (ISG15, IFIT1, IFIT3, MX1, OAS2), Classical_monocyte (S100A8, S100A9, FCGR3B, CSF3R), ACTA2+_monocyte (ACTA2, TAGLN, MYH11, SPARC), cDC1 (XCR1, CLEC9A, FLT3, BTLA, IDO1), and pDC (LILRA4, JCHAIN, MZB1, GZMB) (Figure S4A–C). Among these subtypes, M2_macrophage constituted the largest proportion; its proportion was lower in the TAA group than in controls, although the difference did not reach statistical significance (Figure S4D). Further analyses revealed that CD300A was mainly expressed in ISG+_monocyte, M2_macrophage, and pDC. CLU exhibited a specific high expression in the ACTA2+_monocyte subset. The highest serotonin-related gene set score was observed in Classical_monocyte (Figure S4E).

### 3.10. Expression Confirmation of CD300A and CLU

**Figure 12:**
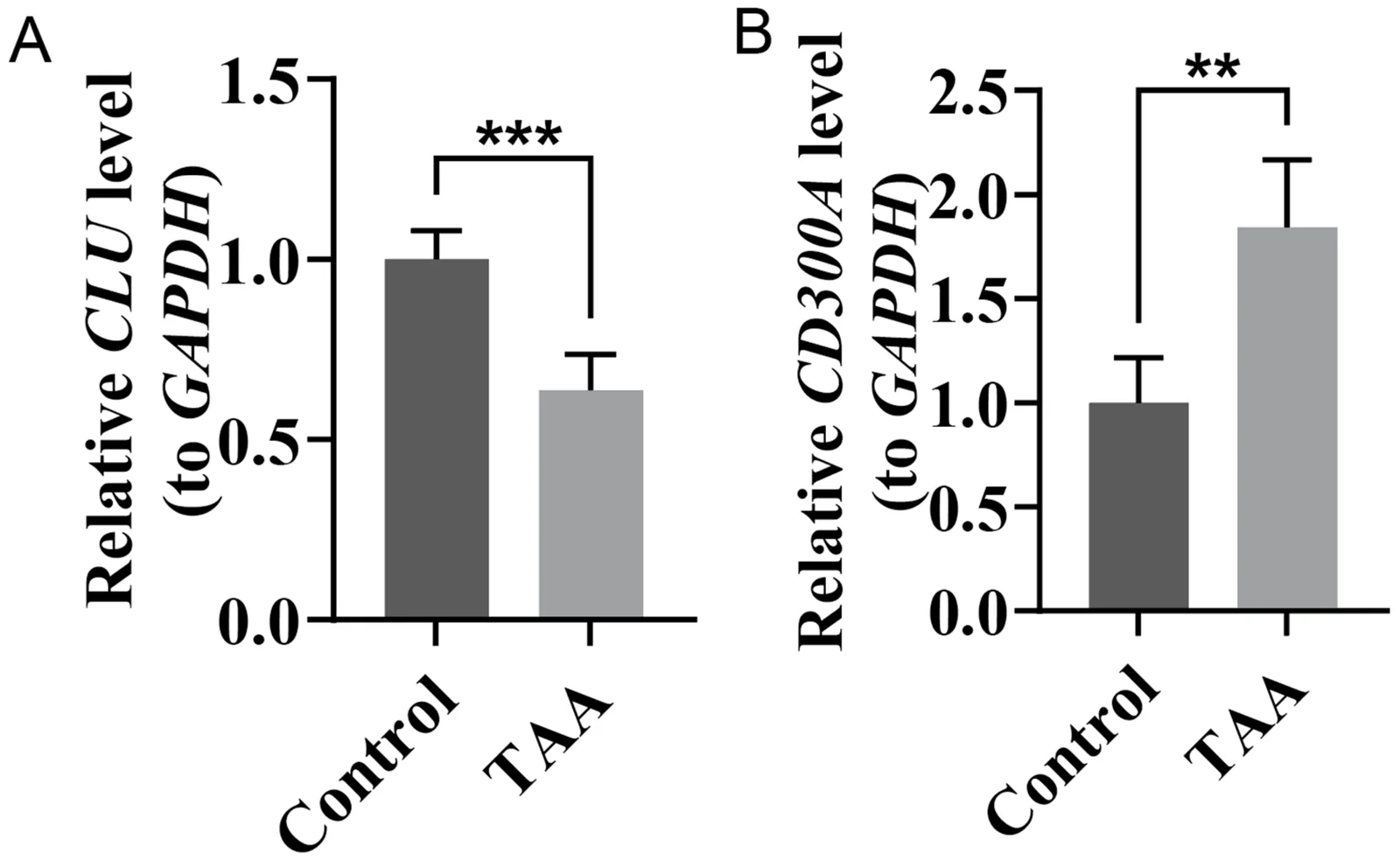
Expression confirmation of CD300A and CLU. (**A**) RT-qPCR analysis showing *CLU* expression in TAA and control tissues. (**B**) RT-qPCR analysis showing *CD300A* expression in TAA and control tissues. TAA, thoracic aortic aneurysm; RT-qPCR, reverse transcription quantitative PCR. ** *p* < 0.01; *** *p* < 0.001.

In RT-qPCR analysis, *CLU* expression in TAA tissues was significantly lower than that in the control group (*p* < 0.01, Figure 12A). Conversely, *CD300A* expression was significantly upregulated in TAA samples (*p* < 0.001, Figure 12B). The consistent expression trends observed using the two methods further highlight their prognostic value in TAA.

## 4. Discussion

Thoracic aortic aneurysm represents a clinically silent yet potentially fatal vascular disease, and the molecular mechanisms underlying its development remain incompletely characterized. Serotonin, long recognized as a vasoactive mediator and immunomodulator, has increasingly been implicated in cardiovascular pathology, yet its specific biomarker-level relevance in TAA has not been systematically addressed. In the current study, we performed an integrated bioinformatics analysis of bulk transcriptomic and single-cell RNA sequencing datasets to identify serotonin-related biomarkers in TAA, and ultimately confirmed two key candidates, CD300A and CLU, as significantly dysregulated molecules with promising diagnostic potential. Notably, our RT-qPCR experiments only verified their expression alterations in TAA tissues and cannot establish serotonin dependence or causal mechanistic roles for these two genes in TAA pathogenesis.

By intersecting bulk-differentially expressed genes (DEGs) with WGCNA-defined serotonin-related co-expression modules, and further refining the candidate list through five machine learning algorithms—LASSO, random forest, SVM-RFE, Boruta, and XGBoost—we identified CD300A and CLU as the consensus serotonin-related biomarkers in TAA. CD300A was significantly upregulated while CLU was significantly downregulated in TAA tissues relative to controls, findings that were reproduced across the independent validation cohort GSE202267 and corroborated by RT-qPCR in clinical tissue specimens. The nomogram constructed from these two biomarkers achieved an area under the ROC curve of 0.830 in the training dataset. Internal bootstrap-correction and ten-fold cross-validation supported its internal stability; nevertheless, owing to limited external sample size, this nomogram remains an exploratory model and requires further validation in larger independent cohorts before clinical risk-stratification application. These results reinforce the value of integrating co-expression network analysis with ensemble machine learning strategies for the discovery of clinically relevant biomarkers in complex cardiovascular diseases [47].

CD300A is an immunoglobulin superfamily receptor that functions as a prototypical inhibitory immune checkpoint, signaling through immunoreceptor tyrosine-based inhibitory motifs (ITIMs) to recruit SHP-1 phosphatase and attenuate downstream immune activation cascades [48]. Expressed on myeloid and lymphoid lineages including monocytes, macrophages, dendritic cells, mast cells, and NK cells, CD300A recognizes phosphatidylserine (PS) and phosphatidylethanolamine (PE) on apoptotic or stressed cells, thereby suppressing cytotoxic immune responses [48]. In the context of malignancy, CD300A-mediated inhibition of NK cell degranulation, perforin and granzyme secretion, and IFN-γ production has been shown to promote tumor immune evasion, and CD300A blockade substantially restores anti-tumor NK cell function in preclinical models [49]. Our data demonstrate that CD300A is markedly elevated in TAA tissues and positively correlated with M2 macrophage and neutrophil infiltration, while inversely correlated with activated NK cell abundance—a pattern consistent with a local immunosuppressive microenvironment in which CD300A-expressing myeloid cells dampen cytotoxic surveillance. To our knowledge, this is the first study to demonstrate the upregulation of CD300A in TAA, and our findings suggest that its aberrant expression may contribute to impaired immune-mediated clearance of damaged aortic wall cells, thereby facilitating disease progression. The link between CD300A and serotonin is further mechanistically supported by evidence that CD300A on mast cells autonomously suppresses IgE-mediated degranulation, and its ablation leads to excessive mast cell activation and markedly elevated serotonin release into local tissue and systemic circulation [50]. This molecular crosstalk implies that CD300A upregulation in TAA may paradoxically contribute to serotonin dysregulation by altering mast cell degranulatory dynamics within the periaortic niche.

Clusterin (CLU), also known as apolipoprotein J, is a widely expressed secreted glycoprotein that functions as a molecular chaperone under cellular stress conditions, suppressing protein aggregation, inhibiting apoptosis, and dampening inflammatory cascades [51]. CLU expression is induced by various cellular stressors including oxidative damage and heat shock, and its down-regulation removes a critical layer of cytoprotective signaling. In the cardiovascular system, CLU has been shown to exert protective effects in cardiac ischemia–reperfusion injury, and its overexpression in murine diabetic atherosclerosis models significantly reduced macrophage pyroptosis, suppressed pro-inflammatory cytokine release (TNF-α, IL-6, IL-1β), and attenuated NLRP3 inflammasome activation, thereby limiting plaque formation and progression [52]. Furthermore, a recent study identified injury-induced CLU-positive cardiomyocytes as critical mediators of macrophage reprogramming toward reparative M2-like phenotypes, illustrating CLU’s broader role in orchestrating cardioimmune interactions [53]. In our study, CLU was significantly downregulated in TAA tissues, which based on the above evidence suggests a loss of its anti-inflammatory and anti-apoptotic protection. The resulting reduction in CLU expression may permit unchecked NLRP3 inflammasome activity, M1 macrophage polarization, and immune imbalance within the aortic wall, collectively accelerating medial degeneration and aneurysmal expansion. The link between CLU and serotonin is mechanistically supported by evidence that CLU mediates anti-inflammatory signaling in the brain by binding brain endothelial cells and suppressing neuroinflammatory gene expression [54], directly implicating CLU as a bridge between immune activation and neurotransmitter system regulation. RT-qPCR validation confirmed that CLU downregulation in clinical TAA tissues was consistent with bioinformatic predictions, affirming its candidacy as a circulating or tissue-level biomarker for disease monitoring.

GSEA analysis revealed that CD300A activity is closely linked to the natural killer cell-mediated cytotoxicity pathway, while CLU was associated with antigen processing and presentation as well as hematopoietic cell lineage enrichment. These pathway associations are biologically coherent with the established roles of both proteins in immune regulation and align with prior evidence that NK cell dysfunction contributes to aortic aneurysm pathogenesis. Histopathological and flow cytometric studies have documented NK cell and iNKT cell infiltration within the human aneurysmal aortic wall, with heightened cytotoxic activity correlating with VSMC apoptosis and elastin degradation [55]. Our immune infiltration analysis further revealed elevated M2 macrophage and neutrophil proportions, alongside decreased Tregs and activated NK cells, in TAA compared to controls—an immune profile consistent with a failure of anti-inflammatory resolution coupled with ongoing matrix destructive activity. CD300A expression was significantly and positively correlated with M2 macrophage and neutrophil abundance, while CLU showed opposing correlations, indicating that the expression of these two biomarkers is tightly intertwined with the immune compositional landscape of TAA. These findings collectively underscore that serotonin-related biomarker dysregulation in TAA is functionally embedded in broader immunological reprogramming of the aortic microenvironment [56].

Immune-driven extracellular-matrix (ECM) degradation represents a unifying pathological hallmark across multiple aneurysm subtypes beyond thoracic aortic disease. Clinical observations in ruptured intracranial aneurysms have reported elevated circulating desmosine, a biochemical marker of elastin breakdown, following aneurysmal subarachnoid hemorrhage; desmosine concentrations correlate with aneurysm size and morphological risk-related features [57]. In abdominal aortic aneurysm, Mordi et al. further demonstrated that plasma desmosine levels were increased relative to controls, correlated with maximal aneurysm diameter, and predicted subsequent AAA-related clinical events independent of baseline aneurysm dimensions [58]. Collectively, these clinical data highlight elastin degradation as a shared downstream readout linking chronic inflammatory activation, ECM remodeling, and progressive aneurysm pathology across distinct vascular territories. In the present TAA study, the myeloid-centered immune perturbations associated with CD300A and CLU fit within this broader conceptual framework: dysregulated MonoMac/DC-mediated inflammatory signaling may promote proteolytic ECM and elastin injury, which in turn fuels aortic wall dilatation and aneurysm progression.

Compound interaction screening via the CTD database identified 2,3,7,8-tetrachlorodibenzo-p-dioxin (TCDD) and bisphenol A (BPA) as the compounds with the highest predicted interaction scores for CD300A and CLU, respectively. Molecular docking confirmed energetically favorable and structurally stable binding: TCDD engaged CD300A at residues P20 and Y21 (−6.6 kcal/mol), while BPA bound CLU at residues D689, E688, and T693 (−7.9 kcal/mol). Notably, these computed binding energies do not support therapeutic potential; docking represents purely in-silico prediction. TCDD, a persistent organic pollutant, activates the aryl hydrocarbon receptor (AhR) to drive oxidative stress, aberrant gene expression, and vascular inflammation [59]. BPA, a widely encountered endocrine-disrupting chemical, has been epidemiologically associated with elevated cardiovascular risk, aortic lipid peroxidation, and arterial dysfunction [60]. Although the in vivo relevance of these interactions in TAA requires further experimental validation, the identification of TCDD–CD300A and BPA–CLU binding interfaces suggests that environmental pollutant exposure may modulate serotonin-related biomarker function and potentially contribute to TAA risk or progression.

scRNA-seq analysis of the GSE155468 dataset identified 11 distinct cell populations within the TAA aortic wall, with monocytes/macrophages/dendritic cells (MonoMac/DC) exhibiting the highest enrichment scores for serotonin-related gene sets among all annotated clusters. This finding positions MonoMac/DC as the primary cellular mediators of serotonin-associated pathological signaling in TAA. Proinflammatory macrophage and DC subpopulations have been consistently identified as key orchestrators of aortic wall remodeling in both experimental and human aortic aneurysm studies, driving VSMC apoptosis and ECM degradation through TNF-α, IL-1β, and MMP secretion [9]. Cell communication analysis revealed that MonoMac/DC interactions with fibroblasts were substantially enhanced in the TAA disease group, with prominent up-regulation of the TNF superfamily signaling axis, the SPP1–integrin axis, and growth factor receptor pathways, collectively reflecting widespread activation of pro-inflammatory, pro-fibrotic, and tissue-remodeling programs. Pseudotime trajectory analysis further revealed a temporal separation of biomarker expression along the MonoMac/DC differentiation continuum, with CD300A expression enriched at the early differentiation stage and CLU expression concentrated at the terminal stage. This sequential expression pattern provides hypothetical mechanistic insight: early-state monocytes bearing high CD300A may establish an immunosuppressive tone by curtailing NK cell and mast cell effector functions, while progressive CLU downregulation at later differentiation stages may remove the anti-inflammatory brake, potentially sustaining inflammation associated with advanced TAA. SCENIC-based transcription factor network analysis identified members of the interferon regulatory factor family (IRF4, IRF5, IRF7) and STAT family (STAT2, STAT5A) as coordinately activated in late MonoMac/DC differentiation subsets, consistent with interferon-driven proinflammatory programming that has been mechanistically linked to aortic disease progression [61]. Virtual knockout experiments using scTeni-foldKnk further demonstrated that perturbation of CD300A and CLU in MonoMac/DC leads to enrichment of distinct downstream pathways: CD300A knockout perturbed genes involved in complement and coagulation cascades and glycolytic metabolism, while CLU knockout disrupted the pyrimidine metabolism and RIG-I-like receptor signaling pathway—suggesting possible non-redundant and complementary regulatory roles in shaping MonoMac/DC function within the TAA microenvironment.

In line with our findings linking myeloid immune dysregulation to aortic inflammatory injury in TAA, excessive inflammation and oxidative stress are well-recognized core pathogenic triggers and negative prognostic factors for cardiovascular diseases and aortic adverse outcomes [62]. Persistent over-activated immune responses exacerbate aortic wall injury, promote extracellular matrix destruction, and accelerate aortic dilatation, thereby increasing the susceptibility to aortic dissection and rupture. Accumulating clinical evidence supports that cardiometabolic interventions with pleiotropic anti-inflammatory and antioxidant properties exert protective and reparative effects on aortic remodeling. Specifically, a recent prospective clinical study demonstrated that GLP-1 receptor agonists are significantly associated with reduced ascending aortic dilatation in diabetic patients, highlighting their promising vascular protective effects beyond metabolic regulation [60]. In this context, circulating inflammatory and oxidative stress biomarkers hold great potential as noninvasive diagnostic, prognostic, and therapeutic monitoring indicators for aortic and cardiovascular diseases.

Several limitations of this study merit acknowledgment. First, our serotonin-related gene set expansion may have introduced potential selection bias, and all bioinformatic analyses were designed primarily for hypothesis generation rather than causal inference. Functional validation through protein-level assays (e.g., Western blotting, immunofluorescence, immunohistochemistry) in larger patient cohorts remains necessary to fully establish the translational significance of CD300A and CLU in TAA. We lacked direct wet-lab functional experiments to verify whether CD300A and CLU exert their effects dependent on serotonin signaling, and we could not establish causal roles of these two biomarkers in TAA pathogenesis. Additionally, the RT-qPCR validation was performed on a limited sample size (n = 5 per group), which, while sufficient to confirm expression trends, limits the statistical power for detecting subtle differences and warrants replication in larger, independent cohorts with diverse clinical characteristics. The constructed nomogram also lacked adequate independent external validation; further external cohort testing is required to confirm its generalizability for clinical risk stratification. Second, molecular docking analyses identified stable binding interfaces between TCDD/BPA and the identified biomarkers, but these computational predictions cannot demonstrate therapeutic potential and require experimental confirmation through binding assays and in vivo exposure studies. Third, pseudotime, CellChat, SCENIC, and virtual knockout analyses applied to the MonoMac/DC compartment were purely in silico computational simulations. These tools can only infer cellular state transitions and molecular associations and cannot confirm true temporal disease progression or causal regulatory relationships. The interaction between TAA pathogenesis and the MonoMac/DC compartment, while supported by scRNA-seq data, would benefit from spatial transcriptomic validation to precisely localize these cellular dynamics within distinct anatomical zones of the aortic wall. Future studies leveraging spatial multiomics platforms will be essential to resolve the spatiotemporal organization of serotonergic immune signaling in TAA.

## 5. Conclusions

In conclusion, this study identifies CD300A and CLU as novel serotonin-related biomarkers in TAA, providing new bioinformatic-derived mechanistic hypotheses regarding how serotonin-associated immune dysregulation—particularly within the MonoMac/DC compartment—may contribute to the chronic inflammatory and remodeling processes that underlie aortic aneurysmal expansion. RT-qPCR only validated their expression trends, and direct functional experiments are required to verify serotonin dependence, causal effects, and therapeutic relevance. The two-gene-based nomogram is an exploratory diagnostic model evaluated by internal bootstrap-correction and cross-validation; reliable external validation in larger cohorts is needed prior to any clinical diagnostic application. These biomarkers show promise for early diagnostic stratification, though their utility as therapeutic entry points remains to be experimentally validated in TAA.

## Data Availability

The datasets analyzed in this study are publicly available in the Gene Expression Omnibus (GEO)(http://www.ncbi.nlm.nih.gov/geo/, accessed on 2 April 2026) repository under accession numbers GSE26155, GSE202267, and GSE155468.

## Supplementary Materials

The following supporting information can be downloaded at: https://www.mdpi.com/article/10.3390/biomedicines14102218/s1, Figure S1. Spearman correlation between biomarkers and original SRGs scores, as well as Bootstrap and cross-validation of the nomogram. Figure S2. Processing of scRNA-seq data. Figure S3. Donor-level cell proportion and biomarker pseudo-bulk expression analysis. Figure S4. MonoMac/DC subtype annotation. Table S1. Serotonin-related genes. Table S2. The sequences of the primers. Table S3. Bulk-DEGs. Table S4. Serotonin-related module genes. Table S5. DE-SRGs. Table S6. Gene Ontology enrichment terms of DE-SRGs. Table S7. KEGG enrichment pathways of DE-SRGs. Table S8. Top 100 DE-SRGs. Table S9. GSEA pathways associated with CD300A. Table S10. GSEA pathways for CLU. Table S11. Correlations between immune cells. Table S12. Number of cells at the donor level. Table S13. Perturbed genes after virtual knockout of CD300A. Table S14. GO enrichment analysis of perturbed genes of CD300A. Table S15. KEGG enrichment analysis of perturbed genes of CD300A. Table S16. Perturbed genes after virtual knockout of CLU. Table S17. GO enrichment analysis of perturbed genes of CLU. Table S18. KEGG pathway enrichment analysis of perturbed genes of CLU. Table S19. Differentially expressed genes in MonoMac/DC. Table S20. GO enrichment analysis of differential genes of MonoMaph/DC. Table S21. KEGG pathway enrichment analysis of differential genes of MonoMaph/DC.

## Abbreviations

5-HT: 5-hydroxytryptamine; AUC: area under the curve; BP: biological process; BPA: bisphenol A; CC: cellular component; DCA: decision curve analysis; DEG: differentially expressed gene; DE-SRG: differentially expressed serotonin-related gene; ECM: extracellular matrix; FC: fold change; FDR: false discovery rate; GEO: Gene Expression Omnibus; GO: Gene Ontology; GRN: gene regulatory network; GSEA: gene set enrichment analysis; GSVA: gene set variation analysis; HVG: highly variable gene; KEGG: Kyoto Encyclopedia of Genes and Genomes; LASSO: least absolute shrinkage and selection operator; MF: molecular function; miRNA: microRNA; MMP: matrix metalloproteinase; MonoMac/DC: monocytes/macrophages/dendritic cells; MSC: mesenchymal stem cell; NES: normalized enrichment score; NK: natural killer; PC: principal component; PCA: principal component analysis; PPI: protein–protein interaction; QC: quality control; RF: random forest; ROC: receiver operating characteristic; RT-qPCR: reverse transcription quantitative PCR; scRNA-seq: single-cell RNA sequencing; SMC: smooth muscle cell; SRG: serotonin-related gene; SVM-RFE: support vector machine-recursive feature elimination; TAA: thoracic aortic aneurysm; TCDD: tetrachlorodibenzodioxin; TEVAR: thoracic endovascular aortic repair; TF: transcription factor; TGF-β: transforming growth factor-β; TOM: topological overlap matrix; Treg: regulatory T cell; UMAP: uniform manifold approximation and projection; VSMC: vascular smooth muscle cell; WGCNA: weighted gene co-expression network analysis; XGBoost: extreme gradient boosting.
