# Supplementary material for: Integrated Analysis of Serotonin-Related Biomarkers in Thoracic Aortic Aneurysm Based on Machine Learning and Single-Cell Sequencing Data
Source: Biomedicines. 2026 Sep 30;14(10):2218. doi: 10.3390/biomedicines14102218 (PMC13701274; doi:10.3390/biomedicines14102218)
Supplement: Supplementary file 1 [file biomedicines-14-02218-s001.zip › Figures.pdf]

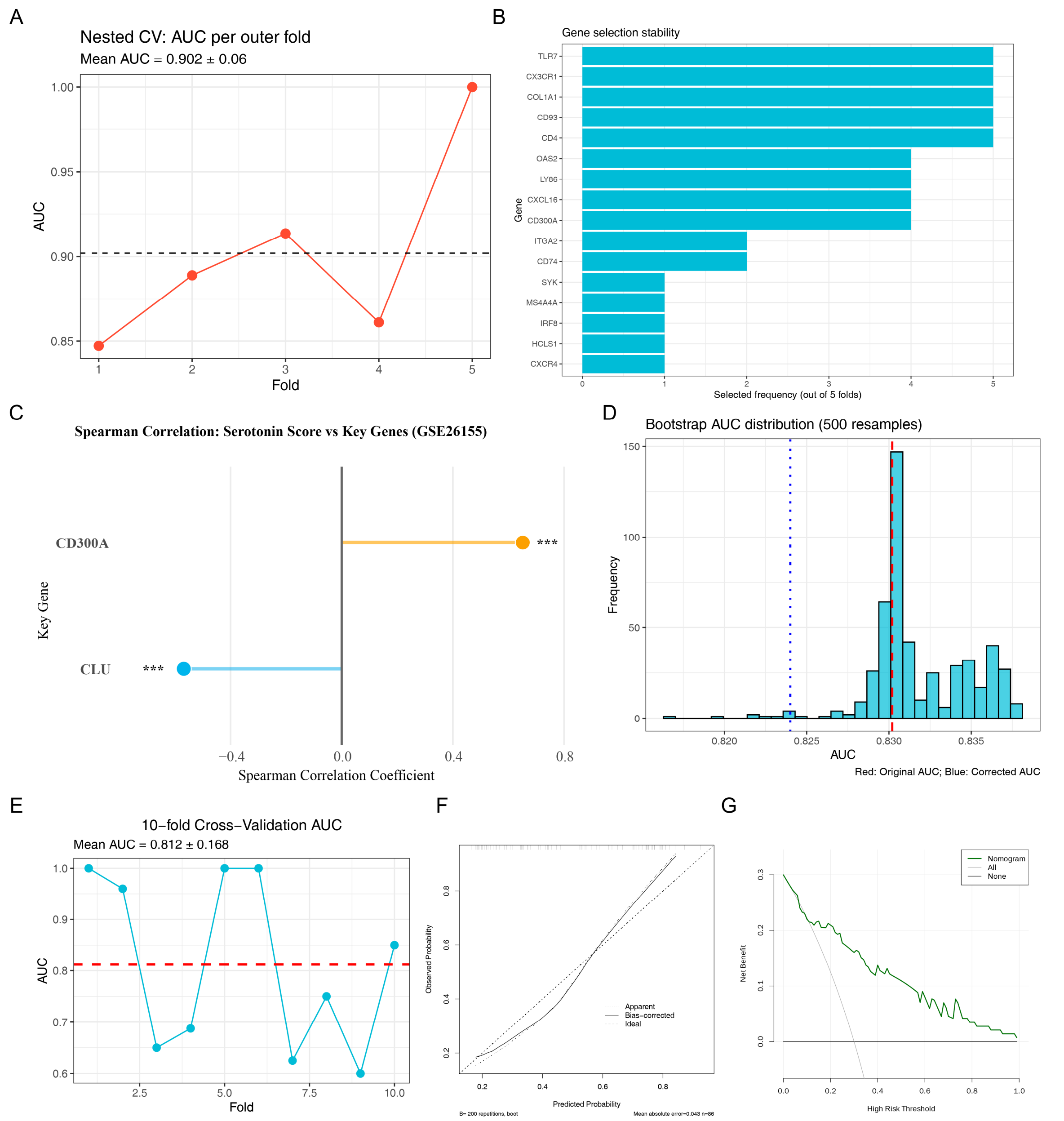

Figure S1. Spearman correlation between biomarkers and original SRGs scores, as well as Bootstrap and cross-validation of the nomogram. (**A**) Spearman correlation between biomarkers and original SRGs scores. (**B**) Bootstrap AUC distribution histogram (500 resampling iterations). (**C**) Ten-fold cross-validation AUC curve plot. (**D**) Bootstrap calibration curve. (**E**) Decision curve analysis (DCA). (F) Calibration curve of the nomogram. (G) DCA of the nomogram. *** $p < 0.001$.

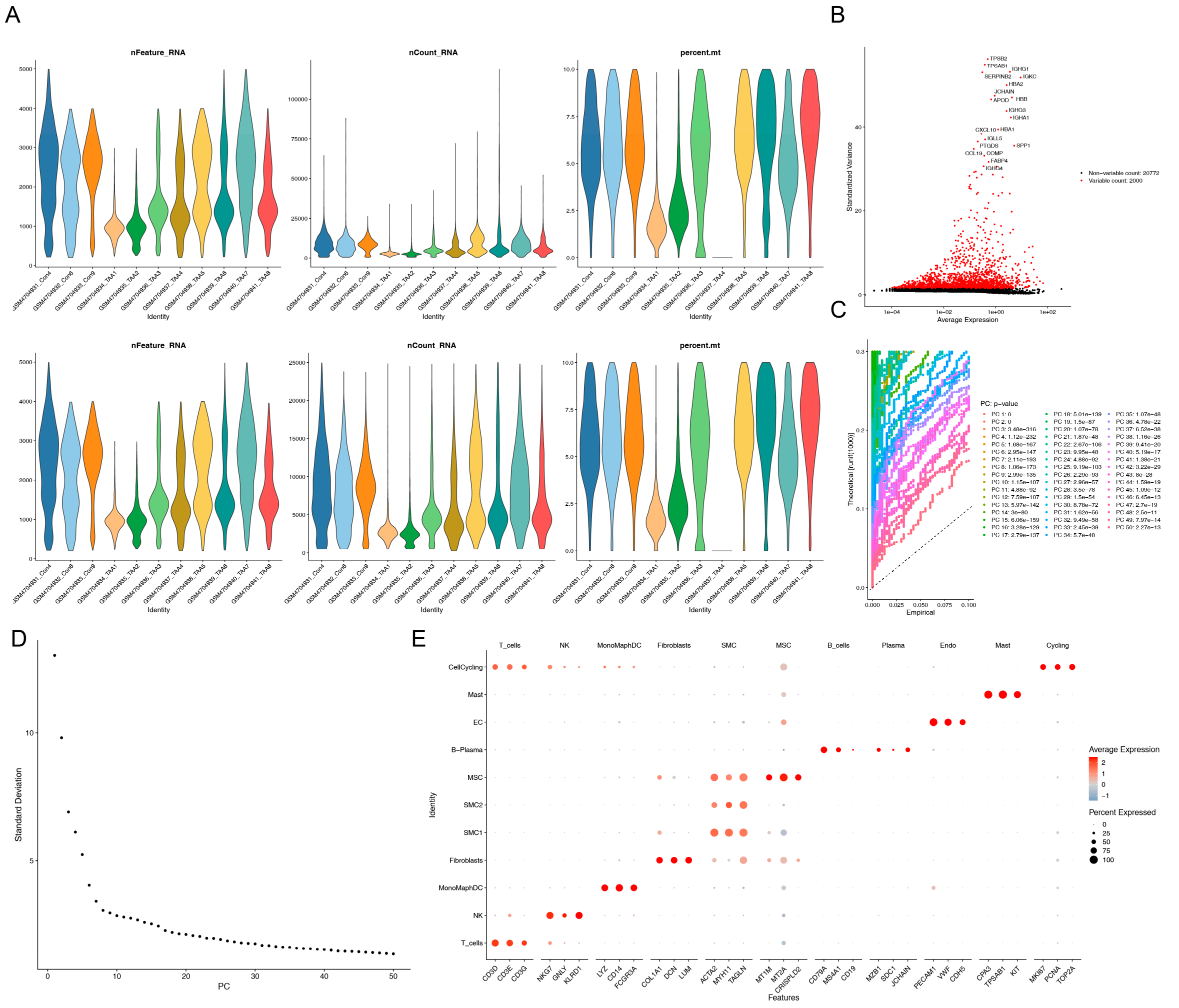

Figure S2. Processing of scRNA-seq data. (**A**) Distribution of raw scRNA-seq data before and after quality control (QC). (**B**) Iden-tification of the top 2,000 highly variable genes (HVGs). (**C**) JackStraw significance plot for principal component analysis (PCA). (**D**) PCA scree plot. (**E**) Expression profiles of cell type-specific marker genes. scRNA-seq, single-cell RNA se-quencing.

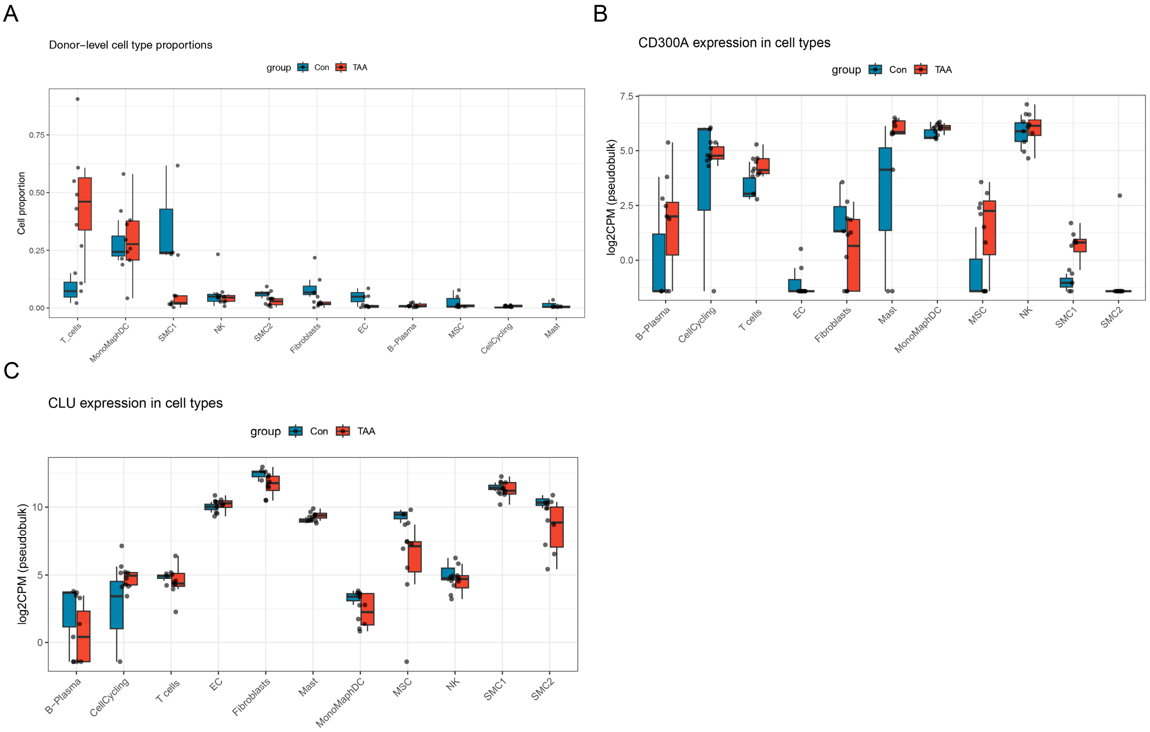

Figure S3. Donor-level cell proportion and biomarker pseudo-bulk expression analysis. (**A**) Percentage of cells at donor level. (**B**,**C**) Biomarker pseudo-bulk expression analysis.

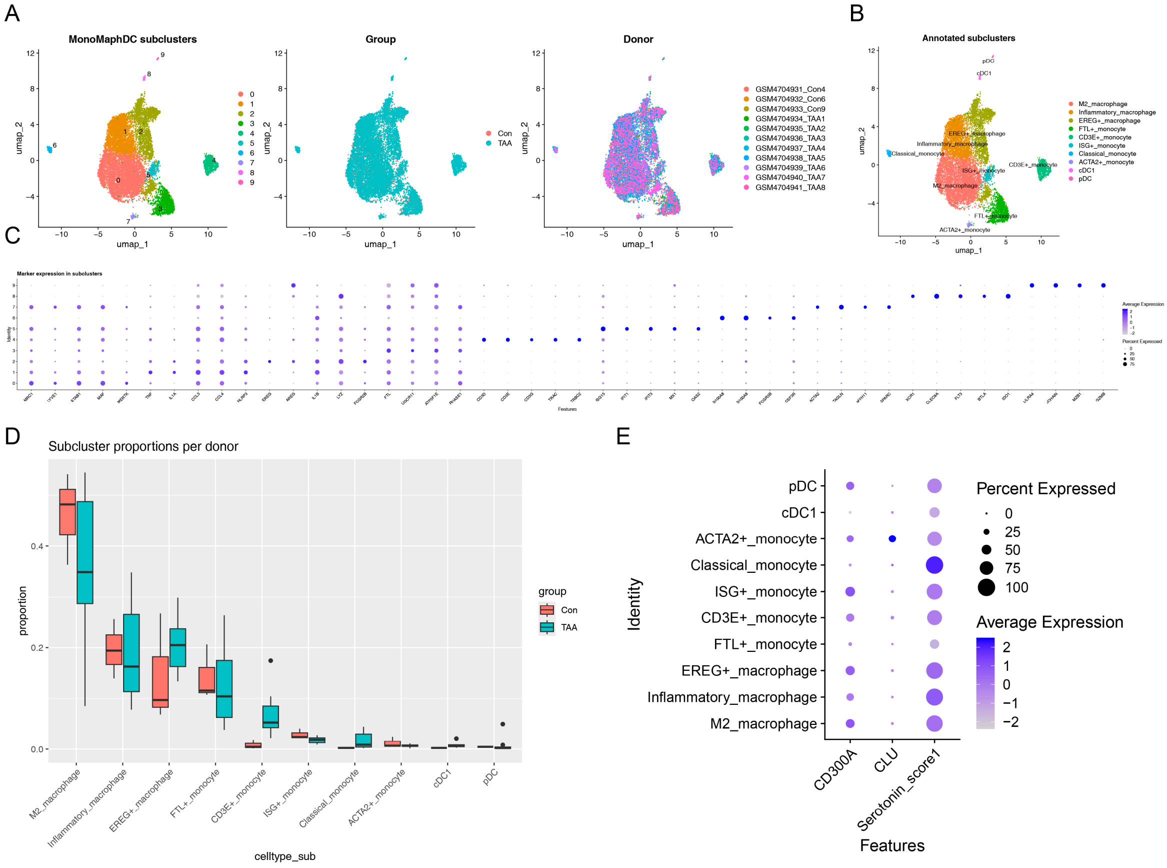

Figure S4. MonoMac/DC subtype annotation. (**A**) Distribution of MonoMac/DC subclusters at the donor level in UMAP plot. (**B**) UMAP plot of subcluster annotation. (**C**) Expression of marker genes in different subclusters. (**D**) Inter-group proportion differences among different MonoMac/DC subtypes. (**E**) Distribution of biomarkers and serotonin scores across different MonoMac/DC subtypes.
